# Interference-driven spacer acquisition is dominant over naive and primed adaptation in a native CRISPR–Cas system

**DOI:** 10.1038/ncomms12853

**Published:** 2016-10-03

**Authors:** Raymond H. J. Staals, Simon A. Jackson, Ambarish Biswas, Stan J. J. Brouns, Chris M. Brown, Peter C. Fineran

**Affiliations:** 1 Department of Microbiology and Immunology, University of Otago, PO Box 56, Dunedin 9054, New Zealand; 2 Laboratory of Microbiology, Wageningen University, Dreijenplein 10, 6703 HB Wageningen, The Netherlands; 3 Department of Biochemistry, University of Otago, PO Box 56, Dunedin 9054, New Zealand; 4 Genetics Otago, University of Otago, PO Box 56, Dunedin 9054, New Zealand; 5 Bio-Protection Research Centre, University of Otago, PO Box 56, Dunedin 9054, New Zealand

## Abstract

CRISPR–Cas systems provide bacteria with adaptive immunity against foreign nucleic acids by acquiring short, invader-derived sequences called spacers. Here, we use high-throughput sequencing to analyse millions of spacer acquisition events in wild-type populations of *Pectobacterium atrosepticum*. Plasmids not previously encountered, or plasmids that had escaped CRISPR–Cas targeting via point mutation, are used to provoke naive or primed spacer acquisition, respectively. The origin, location and order of spacer acquisition show that spacer selection through priming initiates near the site of CRISPR–Cas recognition (the protospacer), but on the displaced strand, and is consistent with 3′–5′ translocation of the Cas1:Cas2-3 acquisition machinery. Newly acquired spacers determine the location and strand specificity of subsequent spacers and demonstrate that interference-driven spacer acquisition (‘targeted acquisition') is a major contributor to adaptation in type I-F CRISPR–Cas systems. Finally, we show that acquisition of self-targeting spacers is occurring at a constant rate in wild-type cells and can be triggered by foreign DNA with similarity to the bacterial chromosome.

## Abstract (web summary)

Prokaryotic CRISPR-Cas systems provide adaptive immunity against foreign nucleic acids by acquiring short, invader-derived sequences called spacers. Here, Staals *et al*. analyse millions of such events in a native CRISPR-Cas system, showing that newly acquired spacers provoke additional rounds of spacer acquisition.

## Graphical abstract

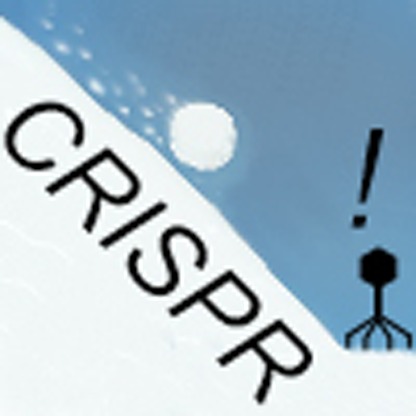

A major determinant for the evolution of microorganisms is the acquisition of foreign genetic elements through horizontal gene transfer^1^,^2^. Since these events can have neutral, beneficial or detrimental effects on host fitness, prokaryotes employ different strategies to balance maintenance and rejection of foreign DNA. One strategy involves the CRISPR–Cas (clustered regularly interspaced short palindromic repeats and CRISPR-associated genes) systems—an adaptive immune system against invaders such as bacteriophages and plasmids^3^,^4^,^5^,^6^. There is considerable diversity in CRISPR–Cas systems, which are divided into two classes, at least six types and 16 subtypes^7^. CRISPR–Cas-mediated defence encompasses three stages: adaptation, expression and interference. During adaptation, exposure to foreign genetic elements results in acquisition of short invader-derived sequences into CRISPR arrays. These arrays are composed of repeats, interrupted by the invader-derived sequences—the spacers. In the expression stage, the CRISPR is transcribed and processed into crRNAs (CRISPR RNAs) that form a ribonucleoprotein complex with Cas proteins (termed Cascade in type I systems)^8^. During interference, the Cas-crRNA complex binds to complementary invader sequences (termed protospacers), resulting in invader degradation, either by recruitment of the Cas3 nuclease (type I systems) or by intrinsic nuclease activity of the Cas-crRNA complex (other types)^3^,^4^.

Our understanding of the molecular mechanism of adaptation is beginning to take shape^9^,^10^,^11^. Despite the diversity of CRISPR–Cas systems, Cas1 and Cas2 domain proteins are present in almost all types^7^. Cas1 and Cas2 are required for adaptation *in vivo*^12^,^13^,^14^ and act via an integrase mechanism to catalyze spacer incorporation *in vitro*^15^,^16^. Proteins with Cas1 and Cas2 domains form complexes^17^,^18^ required for spacer integration, and recently crystal structures of DNA-bound type I-E Cas1–Cas2 complexes were elucidated^19^,^20^. Cas1–Cas2 consists of two Cas1 dimers connected via a central Cas2 dimer (that is, Cas1_2_–Cas2_2_–Cas1_2_) and the distance between Cas1 active sites provides a molecular ruler for spacer measurement. During adaptation, spacers are usually integrated at the leader-end of the CRISPR-array^21^—a sequence including the CRISPR promoter and motifs crucial for integration^13^,^14^,^22^. Integration causes duplication of the leader-proximal repeat^14^ and requires DNA polymerase I and possibly other host enzymes^16^. For type I systems, acquisition of new spacers requires a short protospacer adjacent motif (PAM) that is recognized by Cas1 and Cas2 (refs 14, 19). Type I-E adaptation is RecBCD-dependent and occurs at sites of double-stranded DNA breaks, which commonly occur at replication forks^16^,^23^,^24^. The increased replication of foreign elements, in combination with their paucity of Chi sites that delimit RecBCD activity, appear to explain the acquisition bias toward invader DNA^23^.

The process of adaptation described above is coined ‘naive', since it involves recognition of invaders not previously encountered^10^. Both the PAM and sufficient protospacer complementarity are critical for interference. Phages and plasmids can therefore escape degradation through mutation of these target sequences^21^,^25^,^26^,^27^. Type I-E systems respond to these escape mutants through ‘primed adaptation', whereby new diverse spacers are acquired efficiently in a process requiring Cascade, Cas3 and the crRNA^12^. During priming in the I-E system, new protospacers are predominantly on the same DNA strand of the invader as the priming protospacer (PPS), and there is no apparent locational bias relative to the site of priming^12^,^26^,^28^,^29^. We have previously discovered that priming occurs even when the invader has >10 mutations relative to the pre-existing spacer^26^ and the response is spacer-dependent^30^. This suggests that CRISPR–Cas is robust at removing invaders and that priming is possibly the major adaptation route, even against invaders not previously encountered^26^.

Priming also occurs in *Pectobacterium atrosepticum* type I-F and *Haloarcula hispanica* type I-B systems^31^,^32^. In contrast to type I-E, primed acquisition from a plasmid in the *P. atrosepticum* type I-F system resulted in a similar number of protospacers on either DNA strand and clustering of new protospacers near the primed protospacer^31^. Similar acquisition distributions were observed in the *Pseudomonas aeruginosa* type I-F and *H. hispanica* I-B systems when infected with viruses^32^,^33^. We previously proposed a model for priming by type I-F systems, whereby the Cas-crRNA ribonucleoprotein complex (Csy complex) first recognizes the mutated invader, which leads to the generation of an R-loop and the recruitment of the Cas1:Cas2-3 complex to the displaced (non-primed strand)^31^. Cas1 is essential for adaptation in *P. atrosepticum* and its structure revealed an asymmetric loop that might be unique to type I-F Cas1 proteins^34^. Cas3 helicases are present in type I systems, unwind dsDNA in a 3′-5′ direction and cut the translocating strand via an HD nuclease domain^35^,^36^. We hypothesized that upon encountering a PAM, the translocating Cas1:Cas2-3 complex captures and integrates a new spacer into the CRISPR array. Next, Cas1:Cas2-3 3′–5′ translocation along the displaced (non-primed) strand was proposed to unwind and expose the primed strand, allowing secondary recruitment of Cas1:Cas2-3 and translocation on the primed strand^31^.

Although previous investigations of type I systems have yielded a wealth of data about naive and primed adaptation, these studies have some limitations. First, most studies detect adaptation in strains overexpressing CRISPR–Cas components or in heterologous hosts, possibly leading to non-physiological responses. Second, naive and primed adaptation are rarely investigated using a single experimental strategy. Finally, most studies examine CRISPR expansion within single bacterial colonies or sequence only the spacers that were acquired first by cells in a population—missing multiple incorporation events.

Here, we use *P. atrosepticum* with a native type I-F CRISPR–Cas system to dissect capture and integration dynamics of naive and primed adaptation by sequencing expanded arrays within wild-type populations of millions of cells. We find that priming is >500 times more active than naive adaptation and both processes have no significant difference in PAM preference. Errors occurring during PAM selection correlate with aberrant length spacers and incorrect insertion orientation. We show that the priming site greatly influences the strand and location of targets of new acquisition events: priming typically initiates 5′ of the primed protospacer on the displaced (non-primed) strand. Significantly, the newly acquired spacers (irrespective of whether these were acquired by naive or primed acquisition) strongly influence subsequent capture events, demonstrating that interference stimulates adaptation in a manner stronger than, but similar to, priming. Finally, we observe thousands of natural, yet apparently detrimental, naive and primed acquisition events from the bacterial chromosome, and discover that spacers derived from foreign elements can also stimulate auto-immune self-priming. Taken together, our study allows an unbiased comparison between adaptation from naive and primed targets in a native CRISPR–Cas system.

## Results

### Detection of spacer acquisition in a bacterial population

**Figure 1:**
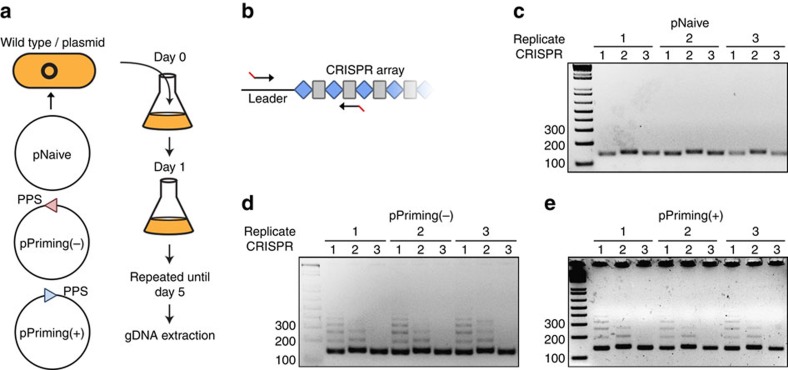
Schematic of the high-throughput spacer acquisition assay. (**a**) Genomic DNA was extracted from wild-type *P. atrosepticum* cells containing plasmids without a protospacer, pNaive, or with a protospacer on either the minus, pPriming(−), or plus strand, pPriming(+), after passaging for 5 days. (**b**–**e**) CRISPR arrays were amplified by PCR and analysed on 3% agarose gels.

To monitor the natural process of naive and primed spacer acquisition in a bacterial population, wild-type *P. atrosepticum* with a plasmid lacking a protospacer (pNaive), or with plasmids carrying a protospacer on either the minus (pPriming(−)) or plus (pPriming(+)) strand were cultured for 5 days without selection (Fig. 1a). These protospacers were complementary to the leader-proximal spacer in CRISPR1 on the chromosome, but carried a non-consensus TG (rather than GG) PAM that triggered priming, as previously observed^31^. *P. atrosepticum* SCRI1043 has a single type I-F CRISPR–Cas system with three CRISPR arrays^37^. CRISPR expansion was assessed by PCR for all three arrays (CRISPR1-3) (Fig. 1b). No expanded arrays were detected for the pNaive cells, suggesting that no, or very few, spacers were acquired (Fig. 1c). In contrast, robust CRISPR expansion occurred in the priming cells (Fig. 1d,e). Therefore, a substantial proportion of the population acquired spacers through priming, whereas naive acquisition was undetectable using this technique.

### Priming is substantially more efficient than naive adaptation

To further analyse the expanded CRISPRs, all PCRs were pooled, enriched for expanded arrays and sequenced on an Illumina MiSeq (Supplementary Fig. 1a,b). Spacers were detected using CRISPRDetect^38^,^39^ and mapped to the plasmid or chromosome using CRISPRTarget^40^ (Supplementary Fig. 1c,d). Over 10 million spacers were acquired in each priming experiment, compared with ∼17,000 in the pNaive samples (Table 1). The diversity of protospacers and arrays observed was high, particularly given the finite number of GG PAMs on the plasmids. The sampling depths were sufficient for the CRISPR populations to be well represented (Supplementary Fig. 2a), and protospacer abundance correlated well with occurrence in unique arrays (Supplementary Fig. 2b).

**Figure 2:**
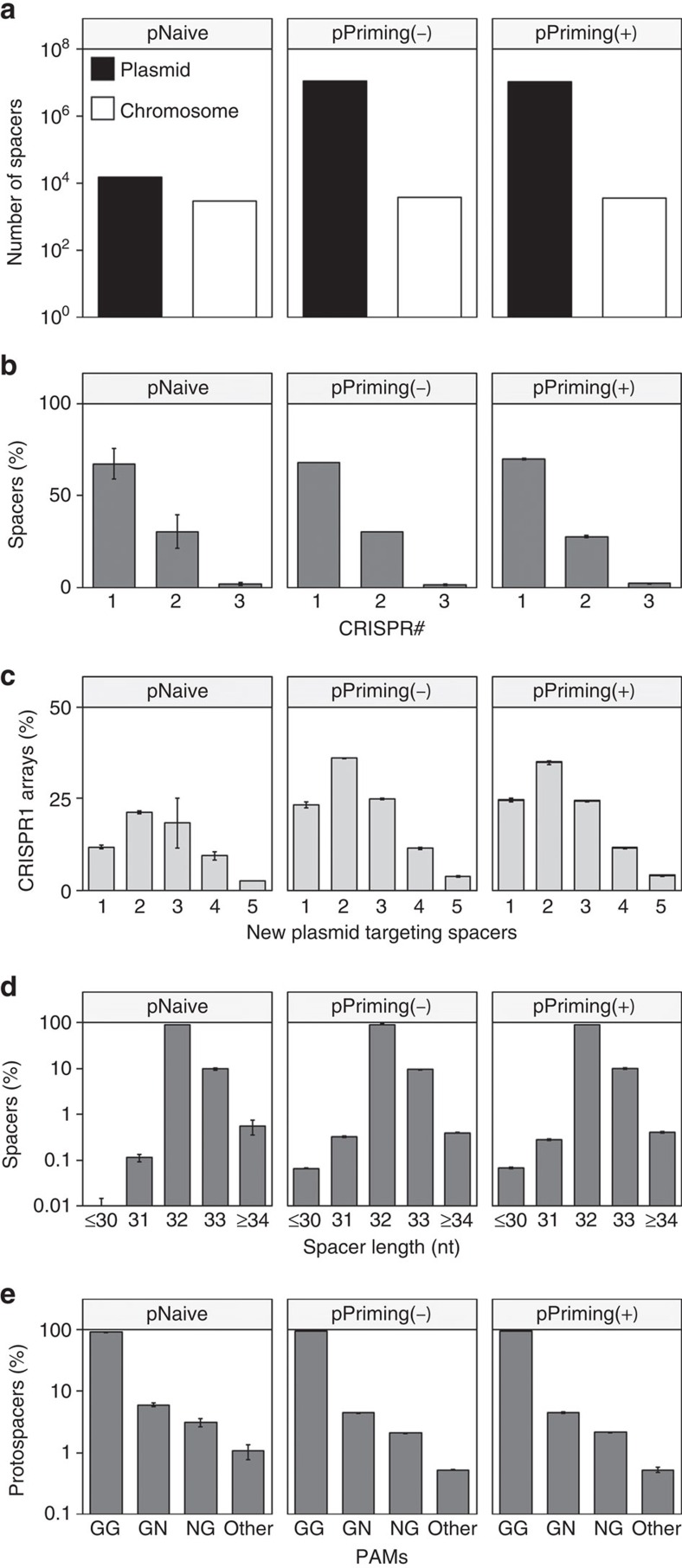
Spacer and protospacer statistics. (**a**) Total number of spacers targeting the plasmid (black) or chromosome (white). (**b**) Proportion of spacers incorporated in CRISPR1, CRISPR2 or CRISPR3. (**c**) Frequency of the number of new plasmid-targeting spacers in CRISPR1. (**d**) Size distribution of new spacers. (**e**) Proportion of protospacers with a GG, GN, NG or other dinucleotide PAM sequence. Note that N stands for every nucleotide excluding G. Error bars represent the s.e. of the mean.

Acquisition from the plasmid was highly favored over the chromosome in all experiments (Fig. 2a). Despite substantial differences in efficiency between naive and primed spacer acquisition from the plasmid, roughly similar numbers of chromosomal spacers were acquired. CRISPR1 had the highest incorporation activity, acquiring ∼70% of new spacers, followed by CRISPR2 (∼30%) and CRISPR3 (∼2%) (Fig. 2b). Most CRISPR1 arrays were expanded by two or more new plasmid-targeting spacers (Fig. 2c), but rarely more than one chromosomal spacer was acquired per array (Supplementary Fig. 3a). Spacers were predominantly 32 nt (∼90%) and 33 nt (∼10%), whereas other lengths accounted for <1% (Fig. 2d). Typical for type I-F systems^41^, 90–93% of all protospacers were flanked by a GG PAM at the 3′ end of the protospacer (Fig. 2e). Interestingly, the majority of protospacers with non-canonical PAMs acquired through naive and primed acquisition contained one G. These experiments provide an unbiased comparison (for example, no Cas protein overexpression) of naive and primed adaptation, and reveal that priming in the type I-F system is >500 times more efficient than naive acquisition. Both forms of adaptation favour 32 nt spacers that target protospacers with 3′ GG PAMs and are biased towards foreign nucleic acids.

### Acquisition errors at canonical PAMs

**Figure 3:**
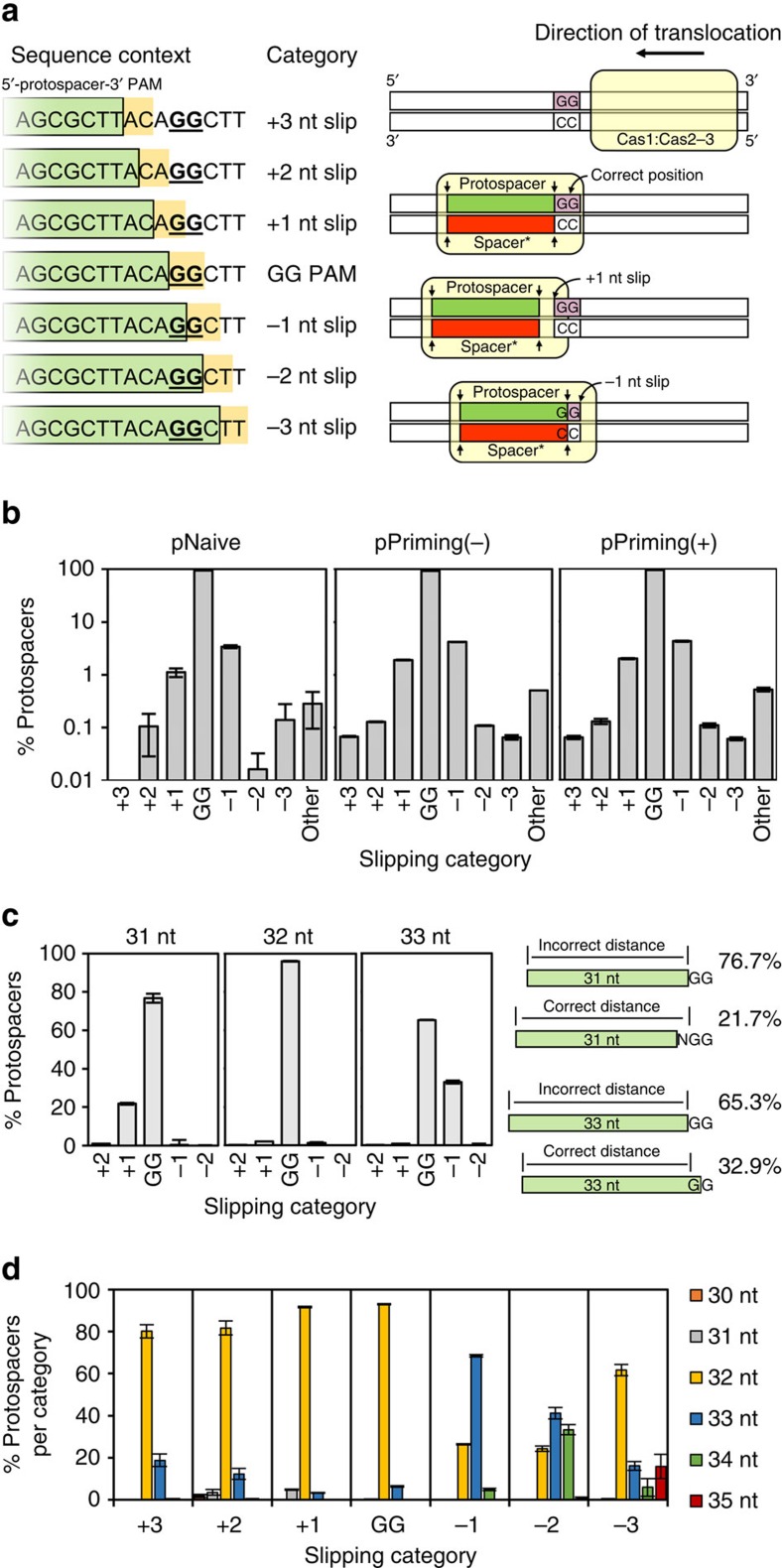
Link between non-canonical PAMs due to slipping and spacer lengths. (**a**) Sequence context and schematic representation of slipping, which results in capture of spacers that map to non-canonical PAMs. ‘Spacer*' denotes the strand with the sequence identical to the spacer in the CRISPR array. (**b**) Frequency of slipping events observed in each dataset. ‘Other' indicates that the 3′ end of the PS was >3 nt from the nearest GG. (**c**) Slipping category assigned to spacers of non-canonical lengths, and schematic view of the mechanistic implications. (**d**) The lengths of spacers resulting from each slipping category. For the analyses in **c**,**d** only protospacers mapping to the plasmid that could not have resulted from flipping are presented. Error bars represent the s.e. of the mean.

Given the prevalence of NG and GN PAMs (Fig. 2e), we examined their sequence context. Typically, the guanine of the NG or GN PAM formed a GG dinucleotide that was either 1 nt distal to, or straddling the 3′ end of the protospacer (Fig. 3a). In cases with other (NN) PAMs, GGs were found ±3 nt of the PAM position for 99.7% of spacers in pNaive and 99.5% during priming (Fig. 3b). This highlights that consensus PAMs are central to spacer capture, and the fidelity of type I-F naive and primed PAM selection does not differ in wild-type cells. We propose that the dominant mechanism of acquisition of spacers with non-canonical PAMs is via ‘slipping' (as previously coined^42^) of the Cas1:Cas2-3 acquisition machinery around these preferred GG locations.

Approximately 0.5% of all protospacers (Fig. 3b ‘other') lacked a GG ±3 nt of the canonical PAM position and showed little PAM bias (Supplementary Fig. 4a). However, the dinucleotide proximal to the 5′ end of these protospacers was enriched for CC, NC and CN (Supplementary Fig. 4b). Acquisition of these protospacers appears to have initiated at a canonical PAM (GG), but the protospacer was subsequently flipped^42^ and integrated into the CRISPR in the opposite direction. The result is a seemingly random PAM, yet with the complementary PAM sequence (in this instance CC, or NC/CN if slipping had also occurred) proximal to the 5′ end of the protospacer. Slipping increases the chance that a spacer flips, since non-canonical PAMs are increased in flipped (Supplementary Fig. 4b; total 53–60%) compared with non-flipped spacers (Fig. 2d; 7–10%). Interestingly, some slips exacerbated flipping—particularly slips of ±2 or more nt (Supplementary Fig. 4c). Therefore, our results indicate that correct positioning of Cas1:Cas2-3 relative to the PAM is required for high fidelity directional integration.

Next, we asked whether aberrant spacer lengths (Fig. 2d) were caused by slipping. Although most 32 nt spacers had GG PAMs (Fig. 2e), comparison of non-canonical spacer lengths and slipping categories revealed that the majority of incorrect length spacers mapped to canonical PAMs (Fig. 3c). Thus, it appears that positional inaccuracy of the PAM distal cut is the predominant contributor to variant length spacers. Examination of the spacer lengths that resulted from slipping (Fig. 3d) revealed that slipping of the acquisition complex past the canonical GG PAM position (+ slips), during capture or integration, results in spacers that are generally measured correctly (32 nt). In contrast, slipping in the opposite direction (− slips) correlates with incorrect length spacers (Fig. 3d).

Since slipping by −1 nt results in incorporation of a G at the 3′ end of the protospacer, other situations that result in a 3′ G might cause 33 nt spacers. Sequential guanine stretches (such as GGG and GGGG) have multiple potential PAMs, but with differing outcomes for the 3′ sequence of the protospacer. For these G-stretches, there was a spacer acquisition bias for 5′ GG pairs (Supplementary Fig. 4d,e), suggesting a preference for non-G nucleotides at the 3′ end of protospacers. However, we did not observe any variation in the spacer-length distributions for spacers with consensus GG PAMs that did or did not include a 3′ G for the protospacer (Supplementary Fig. 4f). This indicates that generation of incorrect length spacers is not promoted by a G at the 3′ end position in the protospacer, such as for the 33 nt spacers observed of −1 nt slipping events (Fig. 3d), but rather the lack of a correctly positioned GG.

### Priming determines location and strand of new acquisitions

**Figure 4:**
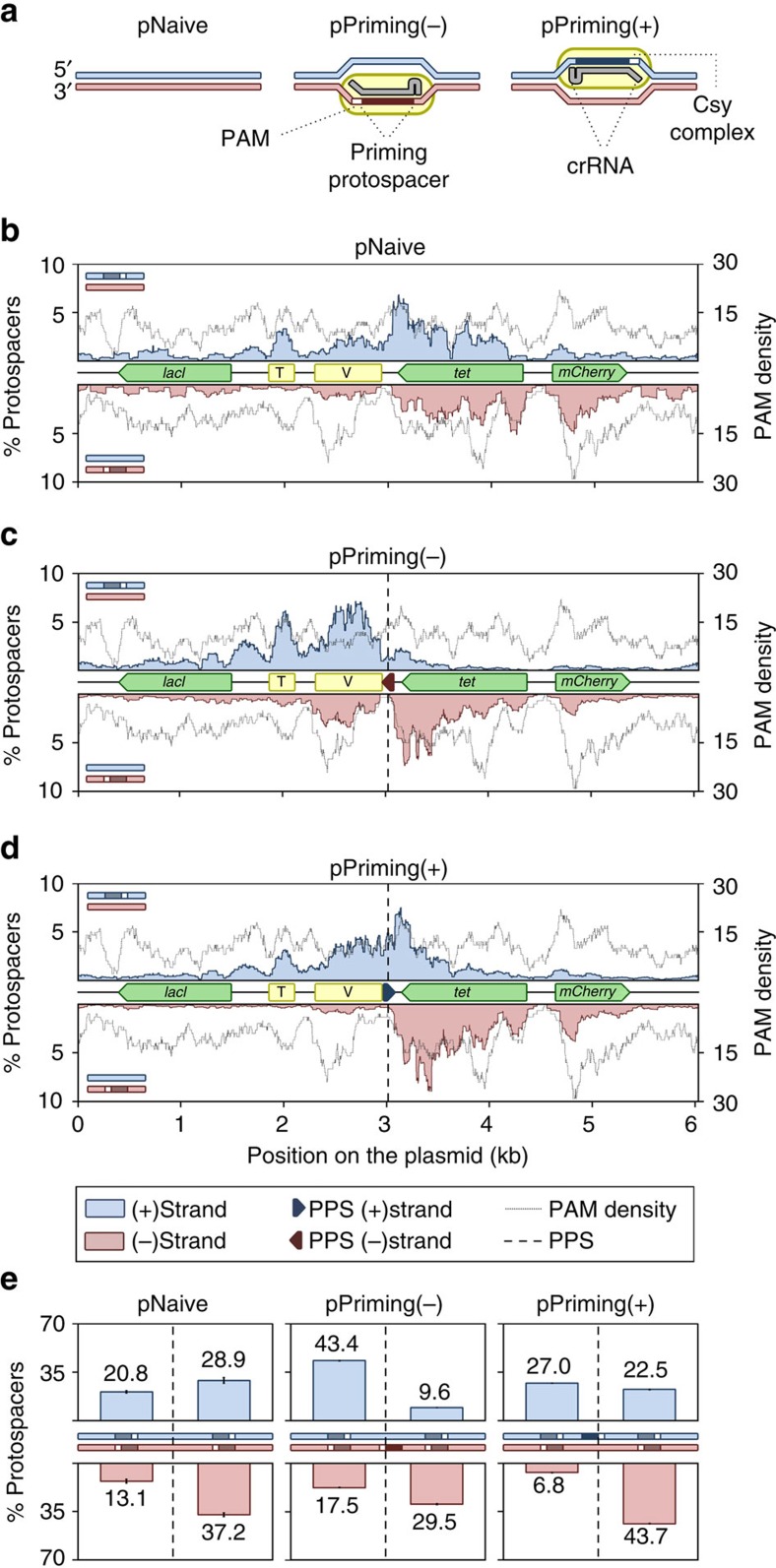
Protospacer mapping. (**a**) Schematic of the experimental setup for initiating spacer acquisition, showing the plus (blue) and minus strand (red) with or without the PPS (dark blue or red, depending on the strand) and PAM (white). The protospacer locations were mapped on (**b**) pNaive, (**c**) pPriming(−) or (**d**) pPriming(+) using a sliding 150 nt binning window. Protospacers on the plus and minus strand are indicated in blue and red respectively. The dotted grey graph in the background depicts the PAM-distribution, which is the density of PAM sequences across the plasmid using a sliding 150 nt binning window. The position of the PPS in pPriming(−) and pPriming(+) is indicated with a vertical dashed line and a dark red or blue arrow, respectively. Genes (green) and other features (yellow) on the plasmids are schematically depicted between the plus and minus graphs. T=oriT (origin of transfer), V=oriV (origin of replication). (**e**) Proportion of newly targeted protospacers 5′ and 3′ of the PPS on each strand. For pNaive this is centred relative to the PPS in the pPriming plasmids. Error bars represent the s.e. of the mean.

We visualized the spacer selection from the plasmids (Fig. 4a) by mapping their protospacer locations. Mapping was remarkably consistent across the replicates (Supplementary Fig. 5). In the pNaive experiment, acquisition occurred throughout the plasmid in a PAM-dependent manner—consistent with the PAM-distribution (Fig. 4b). There was a bias towards transcriptionally-active (*tet*, *mCherry*) and AT-rich regions (oriV and oriT), suggesting that local strand displacement or melting could serve as cues for spacer capture. In addition, the plasmid region encoding the *E. coli* LacI protein was underrepresented.

In contrast to the dispersed distribution in the naive experiment, the primed plasmids displayed very distinct patterns (Fig. 4c,d). For instance, new protospacers from the pPriming(−) experiment mapped close to the PPS (Fig. 4c). Total acquisition from both strands was roughly equal, but a bias for the region 5′ of the PPS on the non-primed displaced (plus) strand was observed (Fig. 4e). In contrast, the protospacer distribution on the primed (minus) strand (Fig. 4c) showed a more uniform distribution (that is, both 5′ and 3′ of the PPS). The distribution was mirrored in the pPriming(+) experiment with the PPS on the opposite strand, confirming this was a priming-specific effect (Fig. 4d,e). Therefore, the PPS greatly influences the location and strand targeted following new capture events.

### Acquisition favors 5′ adenines and thymines in the spacer

To determine if the spacer sequence affected selection and explained the naive distribution, the deviation in the frequency of each base of 32 nt spacers was assessed. Adenines and thymines of the first 5′ nucleotide of the spacer were favored during naive adaptation and priming, and cytosines were strongly underrepresented (Supplementary Fig. 6a,b). This is a common phenomenon in type I-F systems because spacers from species exclusively containing a type I-F CRISPR–Cas system showed a similar trend (Supplementary Fig. 6c). During spacer capture and integration, this bias would translate into avoidance of a 3′ G in the protospacer next to GG PAMs, which is consistent with the preference for 5′ GGs in G-stretches (Supplementary Fig. 4d,e).

### Priming initiates 5′ of the PPS on the non-primed strand

**Figure 5:**
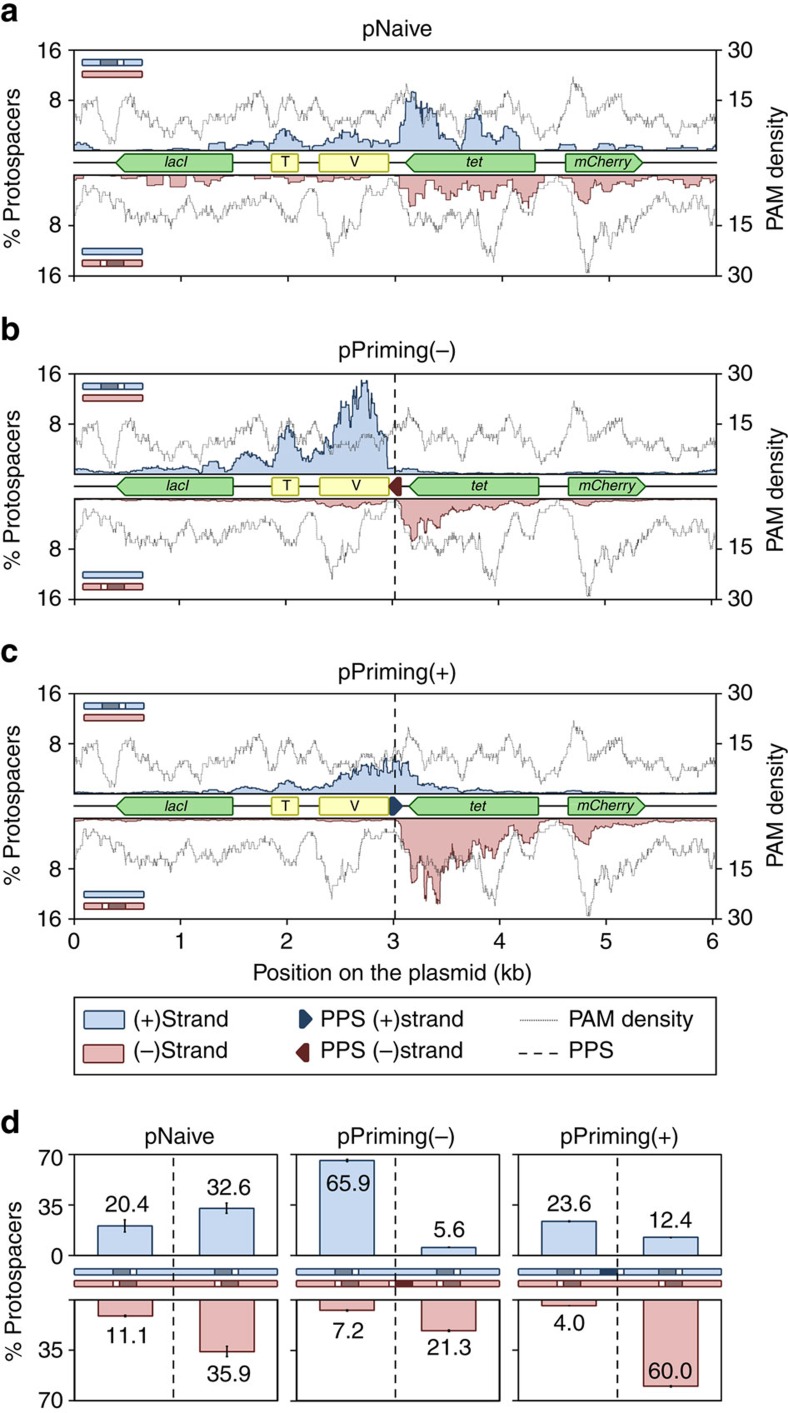
Protospacer mapping of the first acquired spacer in CRISPR1. The protospacer locations of the first acquired spacer in CRISPR1 (that is, the most leader-distal, new spacer) were mapped on (**a**) pNaive, (**b**) pPriming(−) or (**c**) pPriming(+) using a sliding 150 nt binning window. (**d**) Proportion of newly targeted protospacers 5′ and 3′ of the PPS on each strand. Labelling is the same as Fig. 4. Error bars represent the s.e. of the mean.

To explore how priming is initiated, we examined the location of the first spacer acquired. Since newly acquired spacers are usually integrated at the leader-end of arrays^21^, those furthest from the leader and adjacent to the pre-existing spacer, represent the earliest acquisitions (designated herein as S+1). The high incorporation activity of CRISPR1 shows that spacers have a ∼70% chance of being integrated in CRISPR1 (Fig. 2b) and means that the S+1 spacers of CRISPR1 most frequently represent the initial acquisition events. The position of protospacers that these S+1 spacers map to (designated as PS+1 protospacers) was not substantially different from the distribution of all protospacers on the pNaive plasmid (Fig. 5a,d versus Fig. 4b,e). In contrast, for the primed plasmids the PS+1 distribution differed markedly from that of total protospacers (Fig. 5b–d versus Fig. 4c–e), with ∼60–65% of PS+1 located close to, but 5′ of the PPS on the non-primed (displaced) strand (Fig. 5d). These results support a model where priming is typically initiated by Cas1:Cas2-3 recruitment to the non-primed strand of the PPS, followed by Cas3 helicase-driven 3′–5′ translocation and Cas1:Cas2-3-dependent spacer acquisition.

### Newly acquired spacers influence subsequent capture events

**Figure 6:**
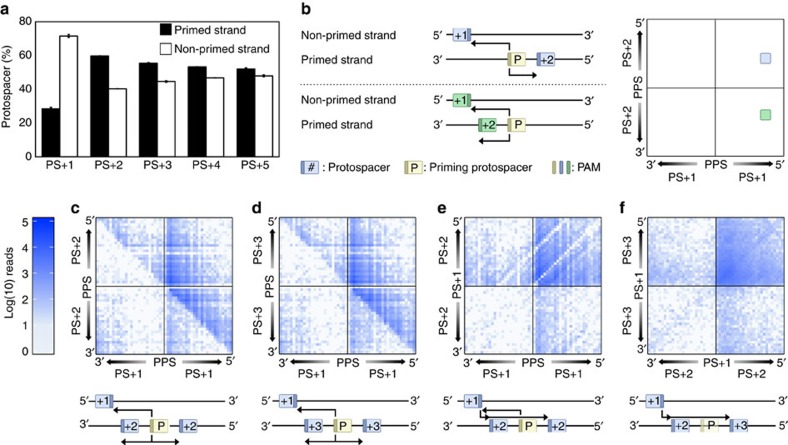
Strand and directionality bias of sequentially acquired spacers. (**a**) Proportion of protospacers targeted by spacers in CRISPR1 mapping to the primed (black) or non-primed strand (white). The protospacer targeted by the first acquired spacer (that is, the most leader-distal, new spacer) is designated PS+1, with later targeted protospacers designated PS+2, PS+3 and so on. Error bars represent the s.e. of the mean. (**b**) Schematic for the interpretation of the heatmaps presented in (**c**–**f**). The (shortest) distance in nt from one protospacer to another was calculated. Protospacers with a 5′ location with respect to a previous one were given a positive distance value, while 3′ located protospacers obtained a negative distance value. The strand used for positive versus negative distance assignment is the strand on which the second protospacer of the pair is mapped. For example, PPS to PS+1 would be scored on the strand that PS+1 was located (that is, the known 3′–5′ directionality of Cas3 on the displaced strand). The distances of two such ‘travels' (for example, PPS to PS+1 and PPS to PS+2) are plotted in the heatmap. Colour-intensity depicts how many reads were associated with these particular ‘travel distances'. Heatmaps were created for the ‘travel distances' from subsequent protospacers on the non-primed (PS+1), primed (PS+2 or PS+3) strands. (**c**) PPS to PS+1 and PPS to PS+2, (**d**) PPS to PS+1 and PPS to PS+3, (**e**) PPS to PS+1 and PS+1 to PS+2, and (**f**) PS+1 to PS+2 and PS+1 to PS+3. The pPriming(−) dataset was used to generate the data displayed in all panels.

We established that most early acquisition events were targeting protospacers (PS+1) on the non-primed strand, 5′ of the PPS (Fig. 5), which is consistent with the known 3′–5′ translocation of type I Cas3 helicases following recruitment to the displaced strand^35^,^36^. In contrast, most subsequent protospacers (PS+2 to PS+5) were on the primed strand (Fig. 6a). Yet, assuming that these later protospacers (PS+2 to PS+5) were also acquired as a result of priming from the PPS, these protospacers (on the primed strand) should also be located 5′ of the PPS. To investigate this, we scored the distance (in nucleotides) and direction (positive values for 5′ and negative for 3′, Fig. 6b) from the PPS to PS+1 (*x*-axis) and from PPS to PS+2 or PS+3 (*y*-axis), weighted by the numbers of associated reads (Fig. 6c,d). As revealed earlier, most PS+1 protospacers were 5′ of the PPS, but protospacers PS+2 and PS+3 were either 5′ or 3′ of the PPS. The lack of 3′–5′ directionality between the PPS and subsequent protospacers prompted us to investigate whether PS+1, rather than the PPS, influenced subsequent acquisitions. Therefore, we analysed the distance from PS+1 to PS+2 and PS+3 protospacers (Fig. 6e,f), which showed that the majority of new PS+2 and PS+3 acquisitions were indeed located 5′ of PS+1. Furthermore, the distances travelled from PS+1 to PS+2 or PS+3, but not from the PPS to PS+2 or PS+3, are consistent with a 3′–5′ translocation model (Supplementary Fig. 7), and not a strand-opening model as previously theorised^31^. We concluded that acquisition of S+2 and S+3 is stimulated by targeting of PS+1 rather than priming from the PPS. This demonstrates that adaptation stimulated by interference-efficient targets is substantially more robust than priming initiated from escape targets. In fact, this effect was so strong that a similar trend was observed for all subsequent protospacers (irrespective of which strands the subsequent protospacers were located on) (Supplementary Fig. 8a-d). Consistently, for the naive experiments, PS+2 and PS+3 protospacers were also influenced by the first spacer acquired during naive acquisition (Supplementary Fig. 9a,b). This mechanism also explains why the expanded array sizes in the naive dataset are comparable to priming, despite the low overall frequency of naive adaptation (Fig. 2a,c). Because the majority of S+1 spacers incorporated during naive or primed adaptation target protospacers with consensus PAMs (Fig. 2d), we propose that newly acquired interference-efficient spacers stimulate the capture of subsequent protospacers.

### Interference promotes spacer acquisition similar to priming

**Figure 7:**
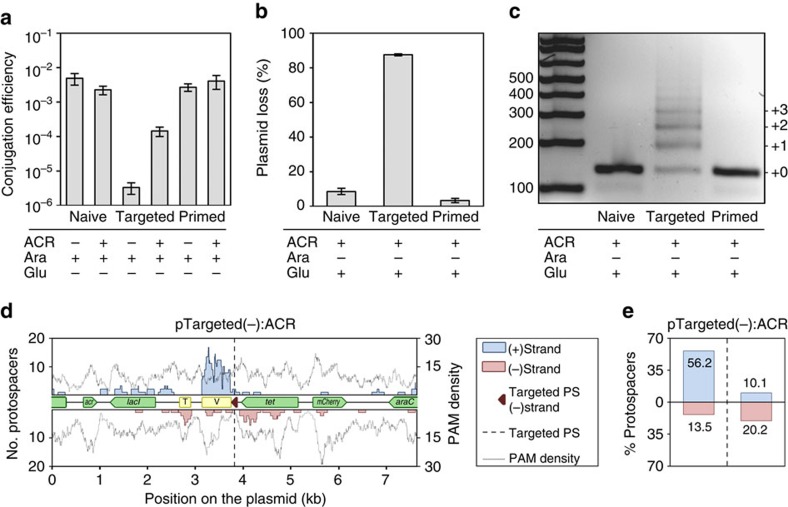
Interference causes spacer acquisition. (**a**) Conjugation efficiencies of the control and anti-CRISPR containing vectors—pNaive:Control, pNaive:ACR, pTargeted(−):Control, pTargeted(−):ACR, pPriming(−):Control and pPriming(−):ACR—into wild-type *P. atrosepticum* in the presence of arabinose (Ara) to induce anti-CRISPR expression. (**b**) Plasmid loss for the transconjugants after 24 h in non-selective media with repression of anti-CRISPR expression by glucose (Glu). Error bars represent the s.e. of the mean. (**c**) PCR of CRISPR1 from pooled culture of the samples in (**b**). (**d**) The protospacer locations of the spacers acquired in CRISPR1 were mapped on pTargeted(−):ACR using a sliding 150 nt binning window. (**e**) Proportion of newly targeted protospacers 5′ and 3′ of the PPS on each strand. Labelling is the same as Fig. 4.

Next, we looked for further evidence that interference-efficient spacers can promote adaptation by examining spacer acquisition from a targeted plasmid. However, targeted plasmids cannot typically be maintained *in vivo* without selection of escape mutations in the PAM, protospacer, *cas* genes or CRISPRs, which complicates analyses of the link between interference and spacer acquisition^30^,^32^,^43^. Therefore, we developed plasmids with an inducible anti-CRISPR gene homologous to AcrF8 from *Pectobacterium* phage ZF40 (refs 44, 45). Anti-CRISPR proteins inhibit CRISPR–Cas interference and/or adaptation^43^,^46^. Naive, primed (TG PAM) and targeted (GG PAM) plasmids were conjugated into wild-type *P. atrosepticum* with expression of the anti-CRISPR. The conjugation efficiency of the targeted plasmid containing the anti-CRISPR was increased ∼50-fold compared with a control lacking the anti-CRISPR (Fig. 7a). This demonstrated that the targeted plasmid underwent CRISPR–Cas interference, and that the anti-CRISPR helped to evade targeting. After 1 day of growth without anti-CRISPR expression the targeted plasmids were rapidly lost, whereas the equivalent naive and primed plasmids were relatively stable (Fig. 7b). Consistent with high plasmid loss (87.5%), extensive CRISPR1 expansion was observed in cells containing the targeted plasmid, but not the naive or primed plasmids (Fig. 7c). The targets of these new spacers were centred around the targeted protospacer (Fig. 7d), with a similar distribution to priming (Fig. 7d,e versus Fig. 4c,e). Thus, interference enhances spacer acquisition in a priming-like manner. These results also show that priming from targeted protospacers is substantially more efficient than acquisition stimulated by primed protospacers (Fig. 7b,c). This is consistent with Fig. 6c–f, which indicated that the first acquired spacer was promoted by the PPS, whereas subsequent spacers were stimulated by the new targeting spacers.

### Primed and naive acquisition of chromosomal spacers

**Figure 8:**
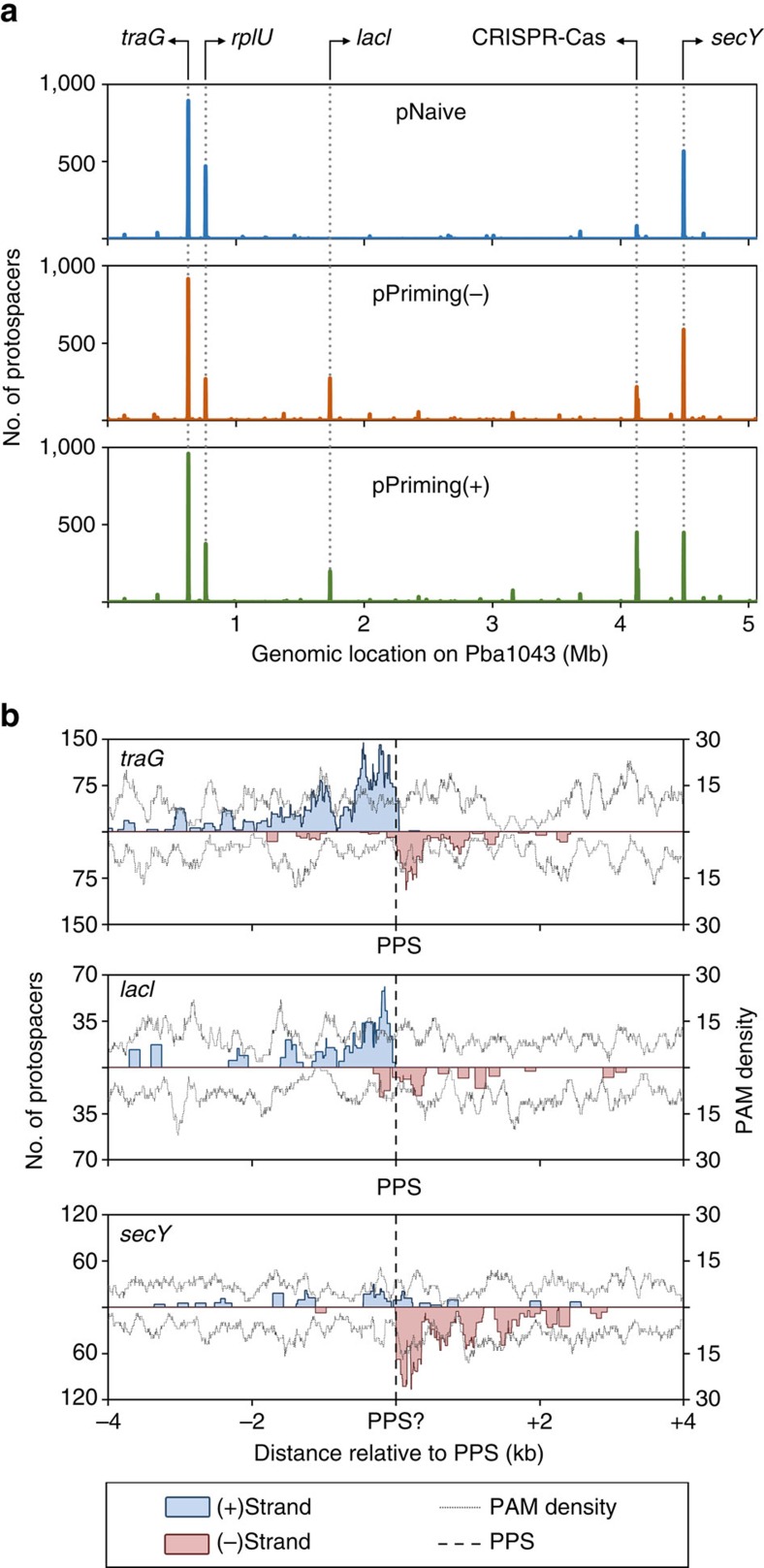
Mapping of chromosomal protospacers. (**a**) Mapping of the chromosomal targeting spacers obtained in the pNaive (upper, blue), pPriming(−) (middle, orange) and pPriming(+) (bottom, green) experiments using a 3,000 nt window sliding in 10 nt increments. Genes at the centre of protospacer ‘hot spots' are indicated on top of the panel. (**b**) Protospacer locations of three chromosomal ‘hot spots' using a sliding 150 nt binning window: *traG* (top), *lacI* (middle) and *secY* (bottom). ‘PPS?' indicates the possible location of an unidentified priming event. Labelling is the same as Fig. 4.

We obtained similar numbers of chromosomal targeting spacers in the naive and primed experiments (Fig. 2a, Table 1). Acquisition generally occurred from similar chromosomal locations between experiments, and the most frequently targeted region was in *traG*, which is part of the pathogenicity island HAI2 (Fig. 8a). Wild-type *P. atrosepticum* contains a spacer in CRISPR2 that perfectly matches a minus strand protospacer with a non-consensus TG PAM within *traG*^47^. Remarkably, the protospacer distribution around *traG* resembled that observed with the pPriming plasmids (Fig. 5), with most protospacers obtained 5′ of the PPS on the non-primed (plus) strand (Fig. 8b). Most of the affected chromosomal regions displayed this priming distribution, as further exemplified by the *lacI* and *secY* regions (Fig. 8b). However, the distributions in the remaining two major cases (*rplU* and CRISPR–Cas) were less evident (Supplementary Fig. 10a,b). These results demonstrate that priming of chromosomal sequences is occurring in wild-type cells. The subsequent self-targeting is likely to result in cytotoxicity or genomic alterations, which, in the case of island-targeting, can include island excision or remodelling^47^. In support of toxicity, the chromosomal spacers were almost always the final spacer acquired within the array (most leader proximal) (Supplementary Fig. 10c) and these arrays rarely acquired more than one chromosomal spacer (Supplementary Fig. 3a). In half of those rare cases where the chromosomal spacer was not the final spacer acquired, its cognate protospacer had a non-consensus PAM, which most likely leads to escape from interference. Overall, for chromosomal protospacers, there were less canonical PAMs (66–70%) compared to plasmid-derived protospacers (93–95%) (Supplementary Fig. 3b)—providing further evidence that these events were detrimental and that non-lethal genotypes were more likely to persist and be sequenced. The rare frequency of self-targeting spacer acquisition that we observed for the priming datasets (∼0.03% of spacers, Table 1) contrasts with the ∼10 fold higher prevalence (0.4%) of such spacers in nature^48^.

Interestingly, self-targeting of *lacI* was common in the presence of priming plasmids, whereas very few *lac*-targeting spacers were detected in the naive acquisition experiment. We theorized that *lacI* self-targeting resulted from primed acquisition from the *E. coli lacI* on the pPriming plasmids. Mapping all spacers of CRISPR1 containing a chromosomal *lacI* targeting spacer (Supplementary Fig. 11a), revealed that many spacers from these arrays were derived from *E. coli lacI* on the plasmid, with one specific spacer present in half of all such arrays. Moreover, this spacer partially matched a *lacI* region on the minus strand of the chromosome, but had 6 mismatches and a non-canonical GC PAM (Supplementary Fig. 11b). The location of this predicted target matched with the protospacer distribution (Supplementary Fig. 11c, Fig. 8b; dashed line), indicating that this spacer was predominantly responsible for priming the chromosome in this region. Taken together, these results demonstrate that self-priming is occurring in wild-type cells and can be triggered by foreign DNA with similarity to the bacterial chromosome. Nevertheless, acquisition of chromosomal spacers is highly counter-selected as it would typically result in cell death^47^,^48^.

## Discussion

**Figure 9:**
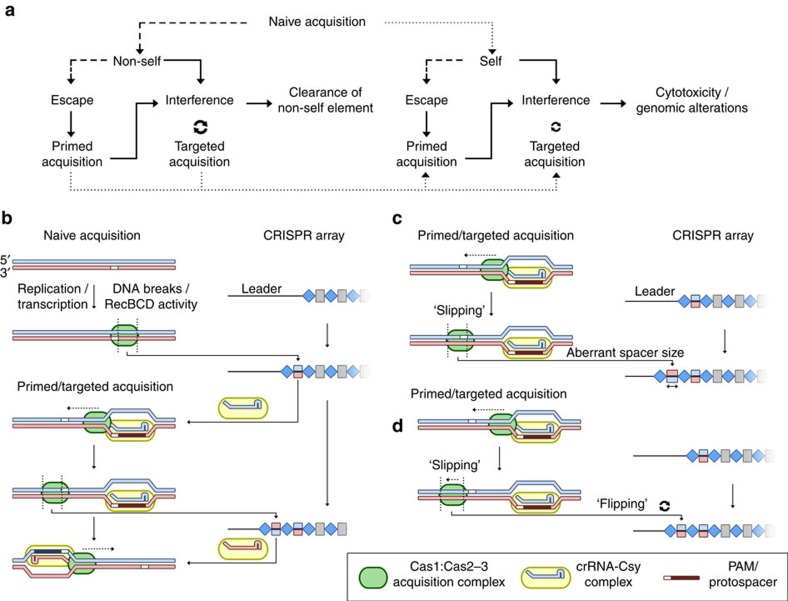
Model for spacer acquisition in the type I-F system. (**a**) Different routes driving spacer acquisition of self and non-self elements. Rare and very rare events are depicted with dashed and dotted lines respectively. Note that most routes converge on the positive feedback loop between interference and targeted spacer acquisition. (**b**) Model for the recruitment and translocation of the adaptation complex. In this example, acquisition of spacers is initiated by naive acquisition, which is quickly followed up by primed or targeted acquisition. The translocations of the acquisition complex are indicated by dotted black lines. Slipping of the acquisition complex upstream of a PAM can result in (**c**) spacers of aberrant length or (**d**) incorrectly-oriented spacers (flipping).

By using a high-throughput spacer acquisition assay in a native type I-F system, we dissected important features underlying naive and primed spacer acquisition, which allowed us to form a new model for adaptation in type I-F systems (Fig. 9). We propose that during naive acquisition, the Cas1:Cas2-3 adaptation complex is recruited to transcriptionally-active regions, stalled replication forks and other features that involve formation of R-loop structures. These criteria often occur on plasmid and phage DNA, resulting in biased acquisition from foreign elements^23^,^49^. Although naive acquisition is inefficient, subsequent interference, resulting from newly acquired spacers, promotes additional adaptation, thus forming a positive feedback loop (Fig. 9a). This probably accounts for the high occurrence of multiple acquisitions (rather than a single, rare event) we observed in the naive setup (Fig. 2c). The feedback loop increases spacer number and diversity, boosting the strength of interference, whilst decreasing the chance of selecting for escape mutants^12^,^28^,^31^. We first speculated that there was a ‘positive feedback loop' in the type I-E system through the generation of acquisition substrates by the Cas3 nuclease^28^. Several additional priming studies have postulated a link between interference activity and spacer acquisition, but priming from escapees potentially formed during the experiments could not be ruled out^28^,^30^,^43^,^50^. We propose that even when escape mutants evade interference through point mutations^25^,^27^, and subsequently trigger primed acquisition, the first new spacer will induce the targeted, interference-linked acquisition response. Our ‘targeted acquisition' model might also explain the protospacer location biases (clustering) observed for population-level spacer acquisition^49^ in other CRISPR–Cas types, where the majority of spacers acquired targeted protospacers with interference-proficient PAMs.

Our evaluation of spacer order, mapping patterns and directional distances led to a new model for the capture of protospacers from foreign DNA during primed and targeted acquisition (Fig. 9b). The Csy complex containing either priming or interference-proficient crRNAs is guided to the protospacer (target strand). The resulting R-loop triggers Cas1:Cas2-3 recruitment to the displaced (non-targeted) strand^17^,^51^. Cas1:Cas2-3 will subsequently translocate in a 3′–5′ direction, scan for PAM sequences, then capture and integrate new spacers into the CRISPR array. We hypothesize that the translocation of Cas1:Cas2-3 is driven by the helicase activity of the Cas3 domain, but this awaits further experimental confirmation^36^,^52^. In rare cases, the acquisition machinery is not correctly positioned relative to the canonical PAM (Fig. 9b,c), resulting in spacers of aberrant size and/or orientation within the array.

As Cas1:Cas2-3 moves along the displaced strand, a GG is detected, presumably by the PAM sensing domain of Cas1 (ref. 19)—but spacer capture efficiency varies for each PAM encountered. The overrepresentation of spacers beginning with A or T means that AGG and TGG locations (or their complement on the opposite strand) are preferred substrates for the I-F system. In type I-E systems, the first nucleotide of the spacer originates from the incorporation of part of the PAM itself, potentially providing a mechanism for directional protospacer integration^12^,^15^,^28^,^41^. Flipping of spacers associated with both canonical and non-canonical PAMs, has been reported in the I-E system, with a higher frequency for the latter^42^. However, type I-F spacers do not incorporate part of the PAM, yet display high accuracy in spacer orientation. Because we only observed extensive faltering of the directional fidelity when PAM slipping by two or more nucleotides occurred, we propose that correct GG positioning relative to the PAM sensing domain in Cas1 confers the directional cue required for integration in the canonical orientation, and that one G in the correct location is sufficient to partially elicit this effect. Akin to type I-F systems, the PAMs of multiple CRISPR–Cas systems include dinucleotides of the same base^41^,^53^, which might allow at least one nt to be sensed appropriately by the adaptation complex during slipping events.

The protospacer bound to the type I-E Cas1–Cas2 complex has a double-stranded helix with splayed single-stranded ends and the branch points are stabilized by two Tyr residues^19^,^20^. The distance between these residues provides a ‘ruler' to determine spacer length. For the I-F system we observed that cuts preceding the PAM (denoted minus slips, given the 3′–5′ translocation of Cas1:Cas2-3) correlated with longer spacers, whereas slipping past the PAM (plus slips) typically resulted in correctly measured spacers. This suggests that for minus slips Cas1:Cas2-3 can re-position itself on DNA after the PAM proximal cut is made, thereby moving the GG into the correct location. Cleavage of the PAM proximal end before the distal cut would be consistent with an integration mechanism where the 3′ (PAM) end of the protospacer performs the first nucleophilic attack^15^. The consequence of an initial PAM proximal cut is that removal of the PAM might displace directionality cues. Our observation that slipping correlates with incorrect spacer orientation, combined with the Cas1–Cas2–protospacer structural symmetry, implies either a coupling between PAM cleavage and integration, or that the structural configuration conferring directionality is retained after both cuts are made, regardless of the order of cleavage. However, the order of cleavage and nucleophilic attack during integration still remains a matter of debate^15^,^54^.

Our approach allowed the first direct comparison between naive and primed acquisition. Primed spacer acquisition is more than 500 times more efficient than naive. In fact, over 5 days, only a tiny fraction of the population underwent naive acquisition. This acquisition was undetectable on gels after array amplification, clearly demonstrating the requirement of deep sequencing to detect such rare events. Caution must therefore be applied in interpretation of previous studies relying solely on gel electrophoresis to conclude naive adaption does not occur. Interestingly, spacers acquired by both naive and primed type I-F adaptation were almost indistinguishable in terms of length, nucleotide composition, and PAMs. This sharply contrasts the reported type I-E bias for selecting non-consensus PAMs during naive acquisition^14^,^29^,^42^,^55^. It is possible that the apparent difference in PAM selection during naive acquisition is due to (over)expression of the *cas1* and *cas2* genes in the type I-E studies, as naive acquisition in the *E. coli* Δ*hns* showed a stronger bias for consensus PAMs^28^. Alternatively, the naive PAM stringency may be specific to type I-F systems, owing to the unique Cas2-3 fusion protein, or the involvement of other Cas proteins^43^. For instance, in type II systems, Cas9 was shown to be essential for spacer acquisition and providing PAM specificity^56^,^57^. The reduced apparent accuracy of PAM identification during naive acquisition for the I-E system might correlate with high plasticity of sequence recognition by the Cascade surveillance complex^26^ and relaxed PAM sensing requirements of Cse1 during interference^58^. Studies in wild-type type I-E systems, similar to our work on type I-F, are required to resolve these questions.

In the naive experiments, protospacers were non-randomly distributed with ‘hot spots' that clustered around transcriptionally-active regions and locations prone to undergo local strand displacement. Interestingly, many spacers were acquired downstream of the origin of replication. The plasmids used have the pMB1 origin of the ColE1 compatibility group, which replicate unidirectionally^59^,^60^. Replication fork stalling on one side of the origin is consistent with the high spacer acquisition we observed in this region^23^. Alternatively, since plasmid replication initiates with an R-loop (not dissimilar to R-loops generated by CRISPR–Cas effector complexes), it is possible that Cas1:Cas2-3 is directly recruited to these regions, as was shown for Cas3 (ref. 61). Although the protospacer distribution during priming markedly differed from naive adaptation, similar ‘hotspots' appeared (oriT, oriV and *mCherry*), suggesting that comparable factors may also contribute to primed spacer acquisition. In addition, for chromosomally-acquired spacers, we predict that transcription and stalling in (replication) forks are major factors driving spacer acquisition. For instance, while we were able to explain the acquisition for the *traG* and *lacI* regions via priming, we could not find pre-existing (or newly acquired) spacers matching *rplU*, CRISPR–Cas and *secY* regions. Interestingly, both *rplU* and *secY* reside in ribosomal gene clusters, which are among the most highly expressed chromosomal regions in *P. atrosepticum* (GEO database accession GSE50468 (ref. 62)). In the CRISPR–Cas region, most protospacers originated near the leader ends of CRISPR1 and CRISPR2, suggesting spacer incorporation and DNA breaks at these highly-active CRISPR arrays contributes to stalling of the replication fork, which promotes new spacer acquisition^23^.

By using two complementary approaches we unambiguously show that both priming and interference initially stimulate the acquisition of spacers close to, but 5′ of, the primed/targeted protospacer on the non-primed/non-targeted strand. We propose that primed and targeted acquisition are in essence similar molecular processes, albeit with different efficiencies. Targeting spacers stimulated both plasmid loss and spacer acquisition more rapidly than priming spacers. This difference might reflect distinct binding modes or affinities of the Csy complex for priming and interference, as recently demonstrated for type I-E Cascade^63^,^64^. In the type I-F systems, both binding modes are likely to recruit the complete Cas1:Cas2-3 adaptation complex^17^, promoting its translocation and the resultant spacer acquisition. It is also possible that a complex of Cas1, Cas2 and Cas3 may form in the other type I systems. For example, in the I-E system, addition of Cas1–Cas2 resulted in Cas3 translocation, and partial reduction of its nuclease activity, when targeting a protospacer with a consensus PAM^65^. Furthermore, the iterative Cas3 recruitments and translocation events away from the site of the protospacer prompted the authors to suggest that priming might occur even in the absence of escape mutations. Therefore, these results support and complement our adaptation data that shows that interference stimulates rapid acquisition of new spacers *in vivo*.

There is a potential downside to primed and targeted acquisition. Acquisition of foreign spacers with partial complementarity to the bacterial chromosome increases the risk of stimulating the acquisition of new interference-efficient spacers, auto-immunity and cell suicide. This might be especially relevant when considering strains containing resident prophages or islands that are infected by related elements. Even a spacer with poor complementary (six mismatches; Supplementary Fig. 11b) initiated priming from the chromosome, suggesting that there is likely a trade-off between specificity and sensitivity in the immune response^26^,^48^. Even in the absence of spacers triggering primed or targeted acquisition, we observed a substantial number of spacers acquired through naive acquisition from the chromosome, particularly in highly transcribed regions. Furthermore, given the lethality that is associated with acquiring self-targeting spacers, the level of naive and primed acquisition from the chromosome we observed is likely to be substantially underrepresented. A study of adaptation in an interference-deficient type II system revealed prolific acquisition of self-targeting spacers^57^, but a higher-throughput study is required to determine if this occurred as a result of ‘self-priming' in a manner analogous to what we observed for the type I-F system. The basal level of self-targeting that occurs at a constant rate in wild-type cells represents a balance between CRISPR–Cas immune functioning and fitness costs for its host. This Achilles' heel of CRISPR–Cas defence has been repurposed to function as a potent anti-bacterial technology^66^,^67^,^68^.

In conclusion, we have extensively characterized the spacer acquisition dynamics of a wild-type CRISPR–Cas system, which has led to a comprehensive model of adaptation in a native bacterium. Adaptation consists of an interconnected feedback pathway of spacer acquisition by naive and primed/interference-associated adaptation. This network is likely to ensure an immune response that can rapidly and robustly respond to foreign elements and their escape mutants.

## Methods

### Plasmids and bacterial strains

Plasmids used in this study (including details of their construction) are given in Supplementary Table 1. Wild-type *P. atrosepticum* SCRI1043 (ref. 69) containing either plasmid pNaive (vector with no protospacer), pPriming(−) or pPriming(+) (vectors with a protospacer complementary to spacer 1 from CRISPR1, but with a non-consensus TG PAM) was grown at 25 °C in lysogeny broth (LB) at 180 rpm. Cells were grown overnight in 5 ml LB and passaged daily for 5 days by transfer of 10 μl to 5 ml fresh LB. Each culture was prepared in triplicate.

### CRISPR array PCRs and preparation of the NGS samples

Roughly 0.5 × 10^9^ cells from the day 5 cultures were used for gDNA isolation using the DNeasy Blood & Tissue Kit (Qiagen). CRISPRs were amplified by PCR using barcoded primers annealing to the leader region of each CRISPR array and secondary primers annealing to spacer 2 of each CRISPR array (primers are provided in Supplementary Table 2). After validating a fraction of the PCR reactions on a 3% agarose gel, all samples were pooled and concentrated by phenol-chloroform- isoamyl alcohol extraction and ethanol precipitation. Expanded CRISPR array amplicons were separated from unexpanded arrays by two rounds of 3% agarose gel purification using the Illustra GFX PCR DNA and Gel Band Purification Kit (GE Healthcare). The resulting sample was analysed on a 2100 Bioanalyzer (Agilent Technologies) before library preparation using the TruSeq DNA Nano Library Preparation (Illumina). To minimize potential biases in read depth of short amplicons compared with longer amplicons, a library of equimolar amounts of amplicons of different sizes was generated in parallel. The pooled libraries were sequenced (2 × 250 base paired-end) on an Illumina MiSeq by New Zealand Genomics Limited (NZGL).

### Data processing and analyses

Sequencing reads were mate-paired and merged using SeqPrep (https://github.com/jstjohn/SeqPrep), using a minimum overlap of 50 nt. Out of the 16,676,264 total read pairs, 15,907,670 (∼95%) were successfully merged. Of these, 84% (13,359,947) had the correct primer-encoded barcodes exactly at both ends of the amplicon. After correcting the orientation, the merged pairs were clustered using 100% sequence identity and length with CD-HIT-DUP^70^, generating 1,770,413 clusters. The clusters were screened with a modified, offline version of CRISPRDetect^38^,^39^, resulting in 1,681,749 clusters with CRISPR arrays containing 6,746,589 spacers (218,572 unique). Spacers were extracted and stored in a FASTA file with a sequence header containing the source read ID and position of the spacer in the array. BLAST databases were created of plasmids pNaive, pPriming(−) and pPriming(+) as well as the genome of *P. atrosepticum* (NC_004547) and used as a reference for protospacer identification by a modified, offline version of CRISPRTarget^40^. A table was created containing a row for each spacer and related information, such as repeat, spacer and protospacer sequence/length/location, 3′ and 5′ sequences flanking the protospacer, CRISPR array, position in the array and so on. Further downstream analyses were performed in Excel and R. One replicate (#2) of the pPriming(−) experiment was omitted from our analysis, since array amplification of the day 1 samples indicated that the population was initially enriched for a clone containing a common spacer that would have biased our analyses.

### Analysis of slipping and flipping

Spacer flipping can contribute to the appearance of apparent non-canonical PAMs; that is, there are ambiguous cases where spacers resulting from either slipping or flipping cannot be differentiated. Therefore, in the analyses presented in Fig. 3 c,d and Supplementary Fig. 4d-f, all spacers that could have resulted from flipping (those where a CC was present within 3 nt of the 5′ end of the protospacer) have been excluded from the analysis; we estimate that overall less than 0.5% of all spacers flipped.

### Bioinformatic analysis of type I-F spacers

Accession numbers from species exclusively containing type I-F specific CRISPR–Cas genes were extracted from a previous study^7^. Spacers were mined from these species and corrected for the right orientation using CRISPRDetect^38^,^39^. The spacer composition (frequency of each nucleotide at each position) of non-redundant, 32 nt spacers was determined (2,316 spacers from 66 different species). The spacer nucleotide composition deviation was calculated by subtracting the observed frequency of each nucleotide from the theoretical normal frequency of 25%.

### Acquisition from targeted plasmids

The anti-CRISPR containing plasmids were constructed as follows. The coding region of the anti-CRISPR (ACR) protein AcrF8 (ref. 45) from *Delftia* sp. 670, accession# KEH13790.1 was sub-cloned from pHERD30T to pBAD30 using EcoRV and HindIII, resulting in the plasmid pBAD:ACR. The region containing AraC and AcrF8 (under P_*araBAD*_ control) from pBAD:ACR was amplified by PCR using PF1764 and PF1765 and cloned into the pNaive, pTargeted(−) and pPriming(−) plasmids, each amplified using PF1763 and PF1766, utilizing NotI and SpeI sites encoded by the primers. Control vectors, without AcrF8, were constructed using the same approach—but beginning with an empty pHERD30T plasmid—to verify function of the anti-CRISPR with our setup. pNaive:ACR, pTargeted(−):ACR and pPriming(−):ACR (plus non-anti-CRISPR containing control plasmids, pNaive:Control, pTargeted(−):Control and pPriming(−):Control) were conjugated from *E. coli* ST18 into wild-type *P. atrosepticum* by filter mating with 0.2% arabinose, which induces expression of the anti-CRISPR, thereby allowing maintenance to the targeted plasmid. Transconjugants were selected by plating onto LB agar+Tc+0.2% arabinose. Colonies from the plates were grown overnight in 5 ml LB+Tc+0.2% arabinose. The resulting cells were washed with phosphate buffered saline and used to inoculate 5 ml LB+20 mM glucose at a 1:1,000 dilution. These were grown for 24 h, then aliquots were diluted and plated onto LB+0.2% arabinose+0.1 mM IPTG. White colonies, which had lost the plasmid harbouring mCherry, were screened for CRISPR array expansion using PCR with primers specific to CRISPR1 (PF174 and PF175). The resulting PCR products were gel extracted and sequenced by Sanger sequencing.

### Data availability

The MiSeq amplicon sequencing data have been deposited in the Sequence Read Archive (SRA) database under accession code SRP074335. Analyses of the data that support the findings of this study are available from the corresponding author upon request.

## Additional information

**How to cite this article:** Staals, R. H. J. *et al*. Interference-driven spacer acquisition is dominant over naive and primed adaptation in a native CRISPR–Cas system. *Nat. Commun.* 7:12853 doi: 10.1038/ncomms12853 (2016).

## Supplementary Material

Supplementary Information Supplementary Figures 1-11, Supplementary Tables 1-2 and Supplementary References

## Figures and Tables

**Table 1:** Number of spacers obtained in the naive and priming experiments per CRISPR array and their respective targets.

	**pNaive**	**pPriming(−)**	**pPriming(+)**
	**# spacers (%)**	**# spacers (%)**	**# spacers (%)**
*CRISPR arrays*			
CRISPR1	12,371 (70.52)	7,523,902 (67.98)	6,965,278 (65.89)
CRISPR2	4,731 (26.97)	3,359,109 (30.35)	3,381,614 (31.99)
CRISPR3	439 (2.50)	185,096 (1.67)	224,208 (2.12)
			
*Spacer targets*			
Plasmid	14,639 (83.45)	11,043,187 (99.77)	10,550,934 (99.81)
Chromosome	2,884 (16.44)	3,790 (0.03)	3,558 (0.03)
Unknown	19 (0.11)	21,131 (0.19)	16,608 (0.16)
Total	17,542 (100)	11,068,108 (100)	10,571,100 (100)
			
*Diversity*			
Unique protospacers*	1,077	9,888	9,751
Unique arrays	1,206	284,781	313,516

^*^Protospacers were grouped based on unique start and end coordinates.
