# Supplementary material for: Interference-driven spacer acquisition is dominant over naive and primed adaptation in a native CRISPR–Cas system
Source: Nat Commun. 2016 Oct 3;7:12853. doi: 10.1038/ncomms12853 (PMC5059440; doi:10.1038/ncomms12853)
Supplement: Supplementary Information — Supplementary Figures 1-11, Supplementary Tables 1-2 and Supplementary References [file ncomms12853-s1.pdf]

Supplementary Figures

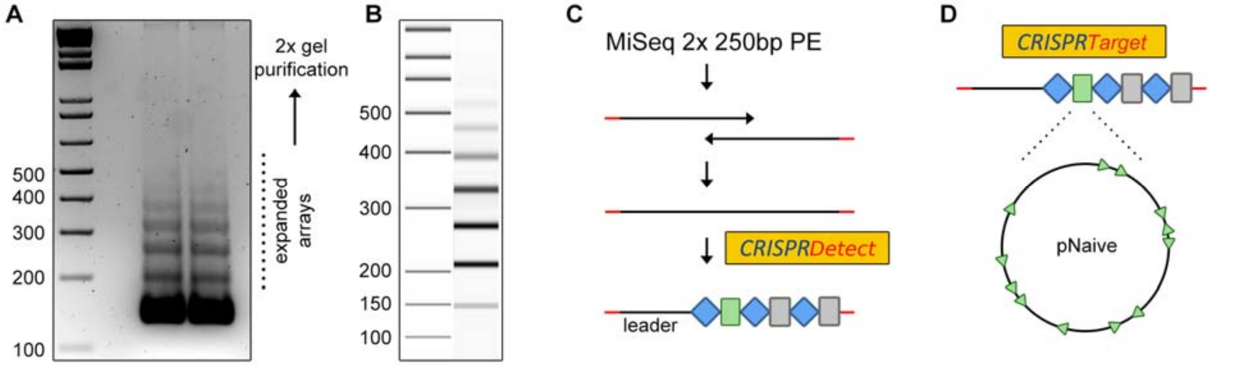

Supplementary Figure 1 | Schematic of the high-throughput spacer acquisition assay after amplification of the CRISPR arrays by PCR. (a) The PCRs from Fig. 1c-e were pooled and expanded arrays were purified from the unexpanded arrays by 2 rounds of gel purification. **(b)** Bioanalyzer result of the purified expanded CRISPR arrays. **(c)** The sample was subjected to 2x 250 bp MiSeq amplicon sequencing and after pair-merging the resulting reads, CRISPR arrays were identified by CRISPRDetect[1]. **(d)** CRISPRTarget[2] was used to map the spacers and to extract other relevant information.

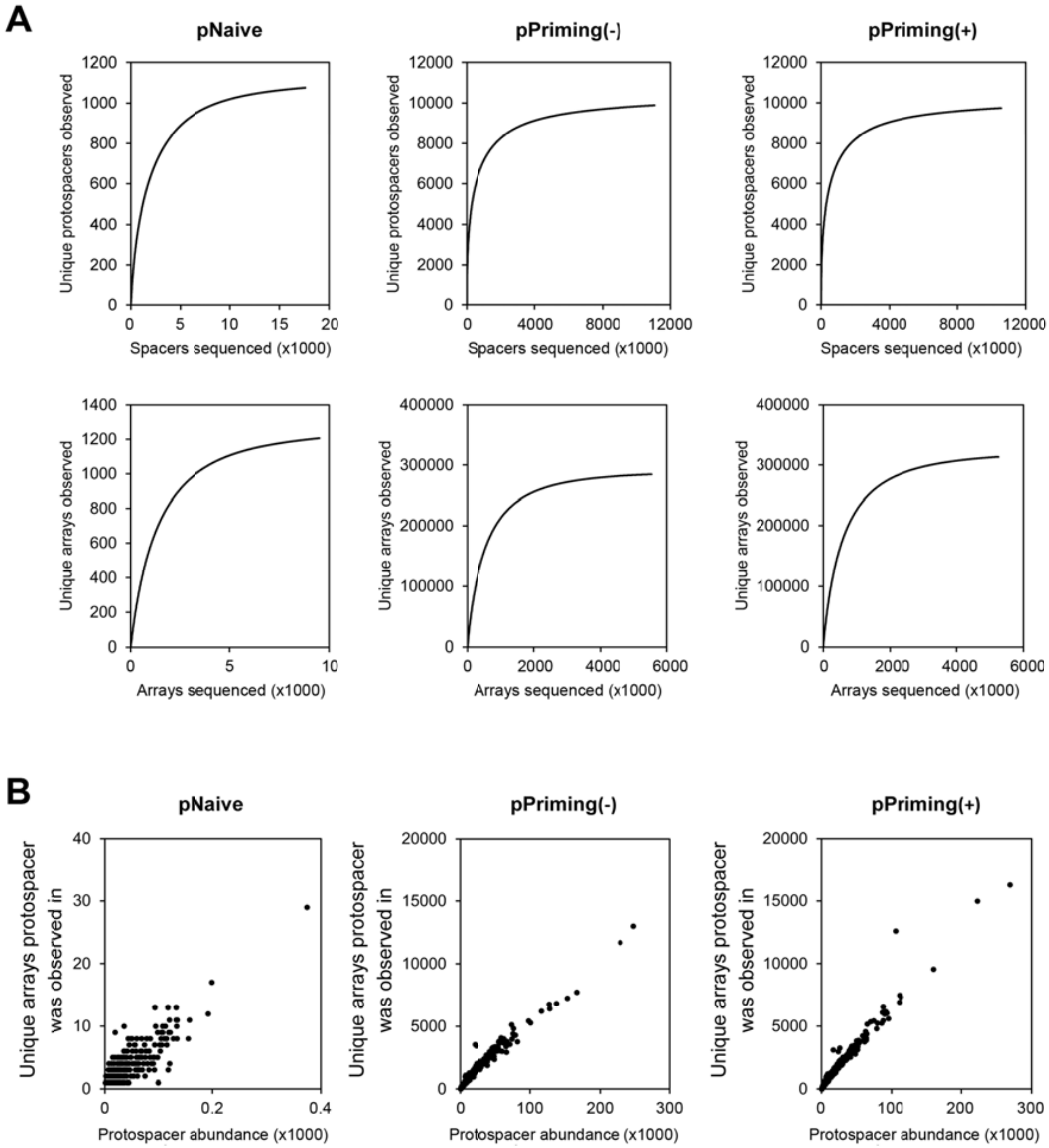

Supplementary Figure 2 | Rarefaction curves for spacers and arrays. (a) Unique protospacer observation as a function of the number of spacers sequenced (upper panels), and unique arrays (i.e. distinct combinations of (proto)spacers forming arrays) observed as a function of the number of arrays sequenced. **(b)** Correlation between protospacer abundance and frequency of occurrence in unique arrays. To avoid artificial inflation, resulting from sequencing errors, of the data presented in (a and b), we have based these diversity statistics on unique protospacers rather than unique spacers.

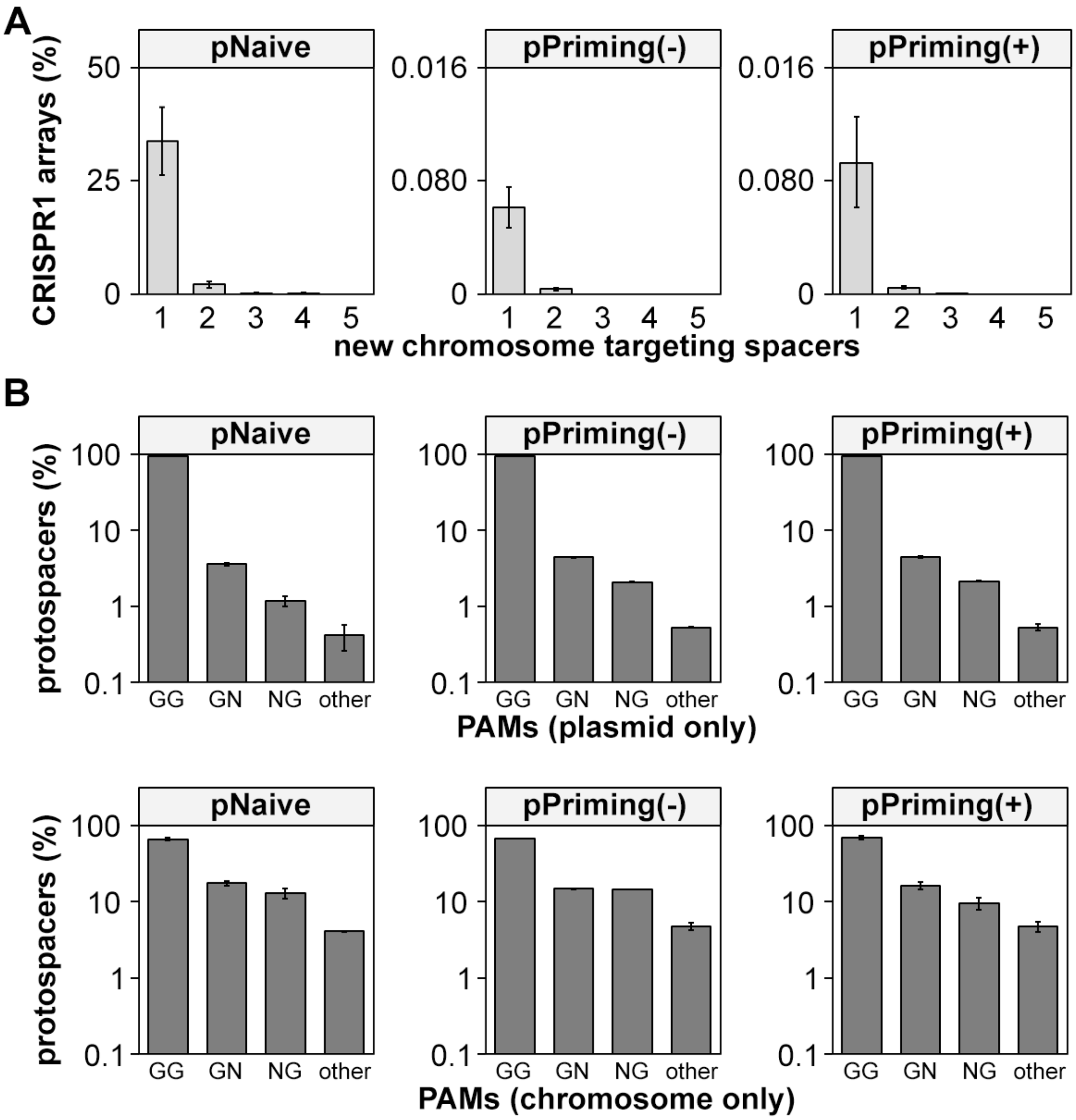

Supplementary Figure 3 | Characteristics of self-targeting spacers compared to plasmid-targeting ones. (a) Frequency of the number of new chromosome spacers in CRISPR1. **(b)** Proportion of protospacers on the plasmid (top) or chromosome (bottom) with a GG, GN, NG or other dinucleotide PAM sequence. Note that N stands for every nucleotide excluding G. Error bars represent the standard error of the mean.

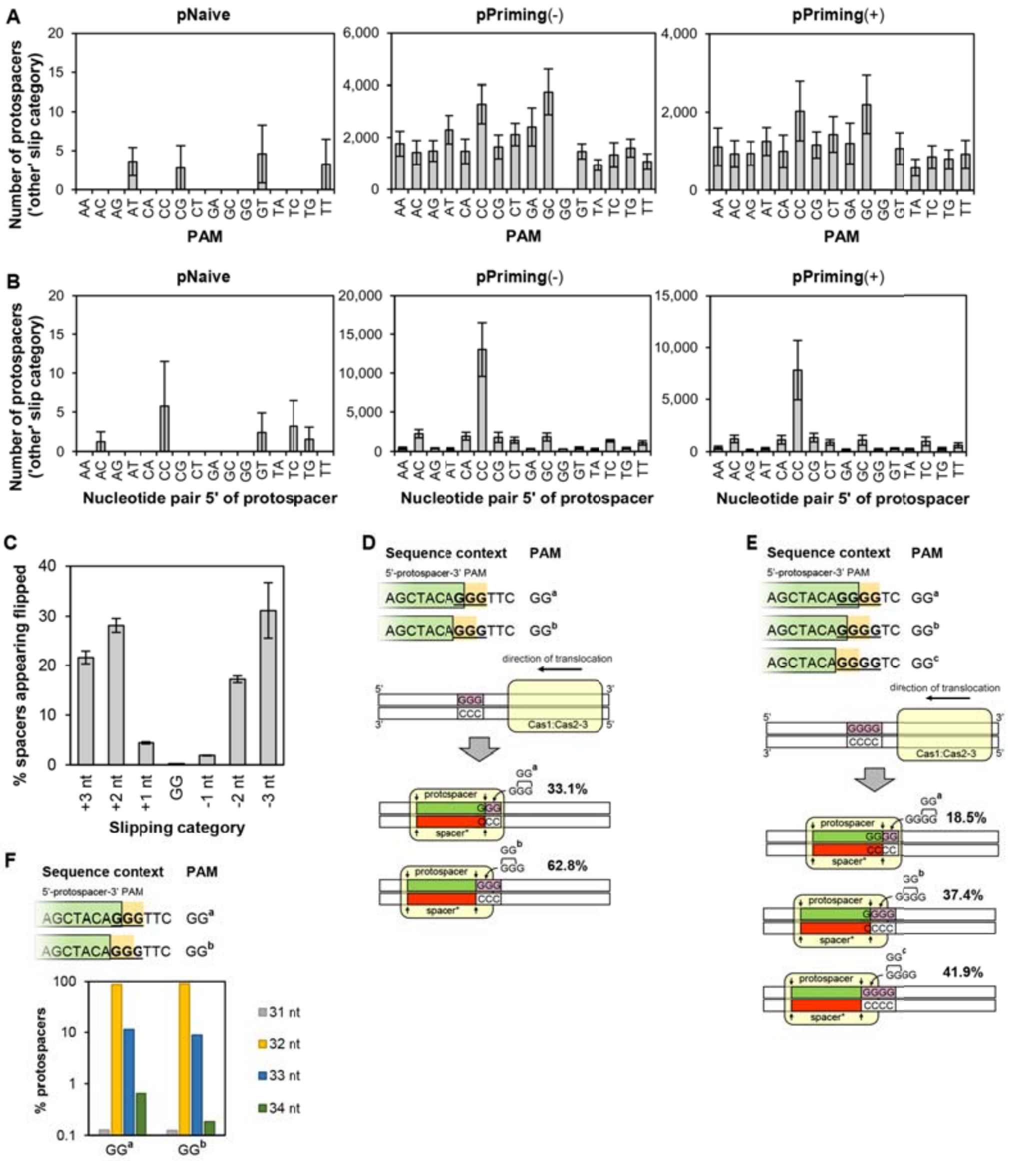

Supplementary Figure 4 | Non-canonical PAMs, slipping and flipping. (a) The PAM frequencies for protospacers mapping to the plasmids whose 3’ ends were > 3 nt from the nearest GG. **(b)** Frequency of the nucleotide pair found proximal to the 5’ end of protospacers from the ‘other’ category in Figure 3b; for pNaive the reads are low (<50), whilst pPriming(-) and pPriming(+) each represent >50,000 reads. **(c)** Flipping frequency of each slipping category. **(d)** Sequence context and schematic of acquisition from GGG stretches for protospacers with GG PAMs. **(e)** Sequence context and schematic of acquisition from GGGG stretches for protospacers with GG PAMs. **(f)** The lengths of spacers with GG PAMs that resulted from acquisition at GGG stretches. Error bars represent the standard error of the mean.

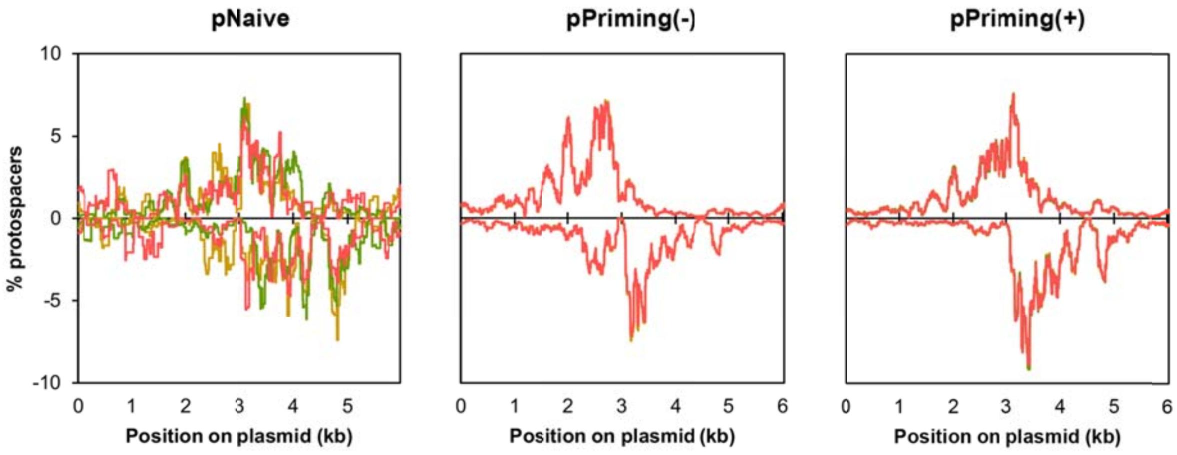

Supplementary Figure 5 | Protospacer mapping per replicate. The protospacer locations from each of the replicates (in green, yellow and red) were mapped on pNaive, (left) pPriming(-) (middle) or pPriming(+) (right) using a sliding 150 nt binning window.

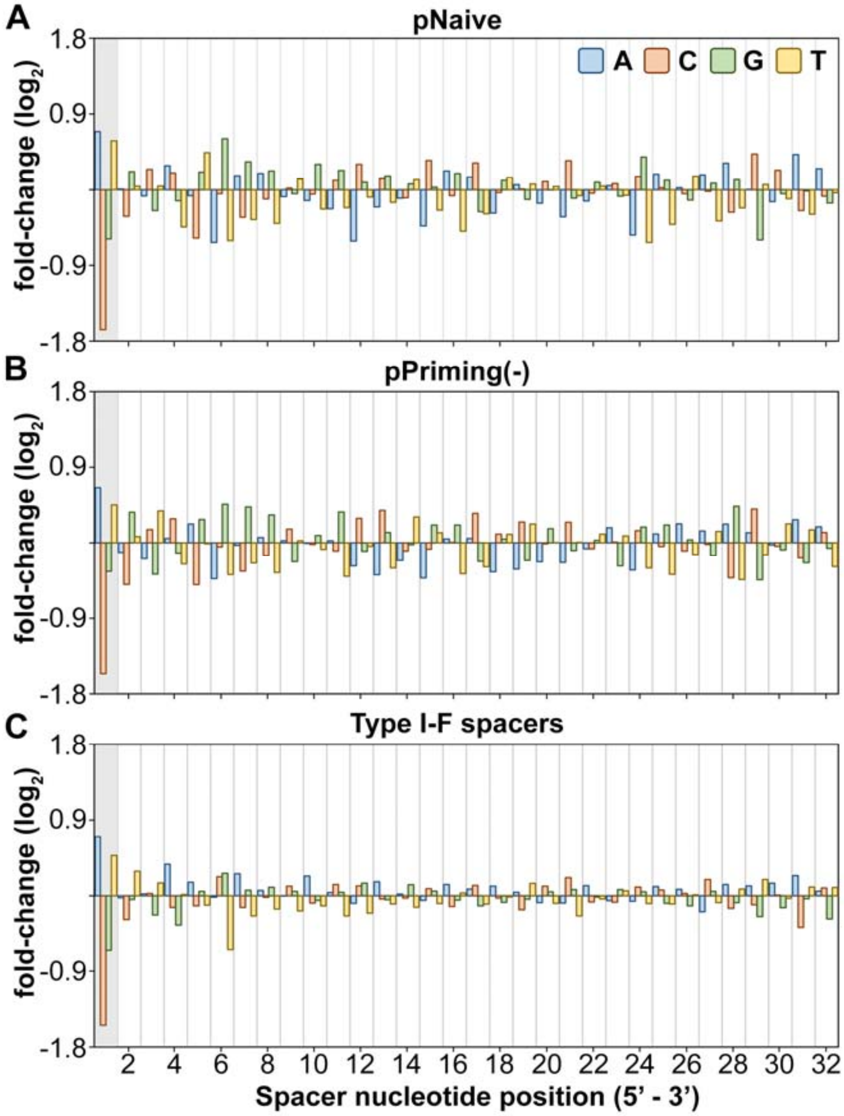

Supplementary Fig. 6 | Spacer nucleotide composition. The nucleotide distribution of new 32 nt spacers with GG PAMs in the **(a)** pNaive or **(b)** pPriming(-) experiment was determined for each spacer nucleotide position (with position one being the most 5' nt of the spacer) and was compared to the normal distribution (i.e. all sequences 5' of GG dinucleotides) on these plasmids. The y-axis shows the difference between these distributions (observed – normal). **(c)** Similar analysis of 32 nt spacers extracted from species exclusively containing a type I-F CRISPR-Cas system (2316 non-redundant spacers from 66 species). Since it was not possible to determine the normal distribution, a frequency of 25% was assumed for each nucleotide.

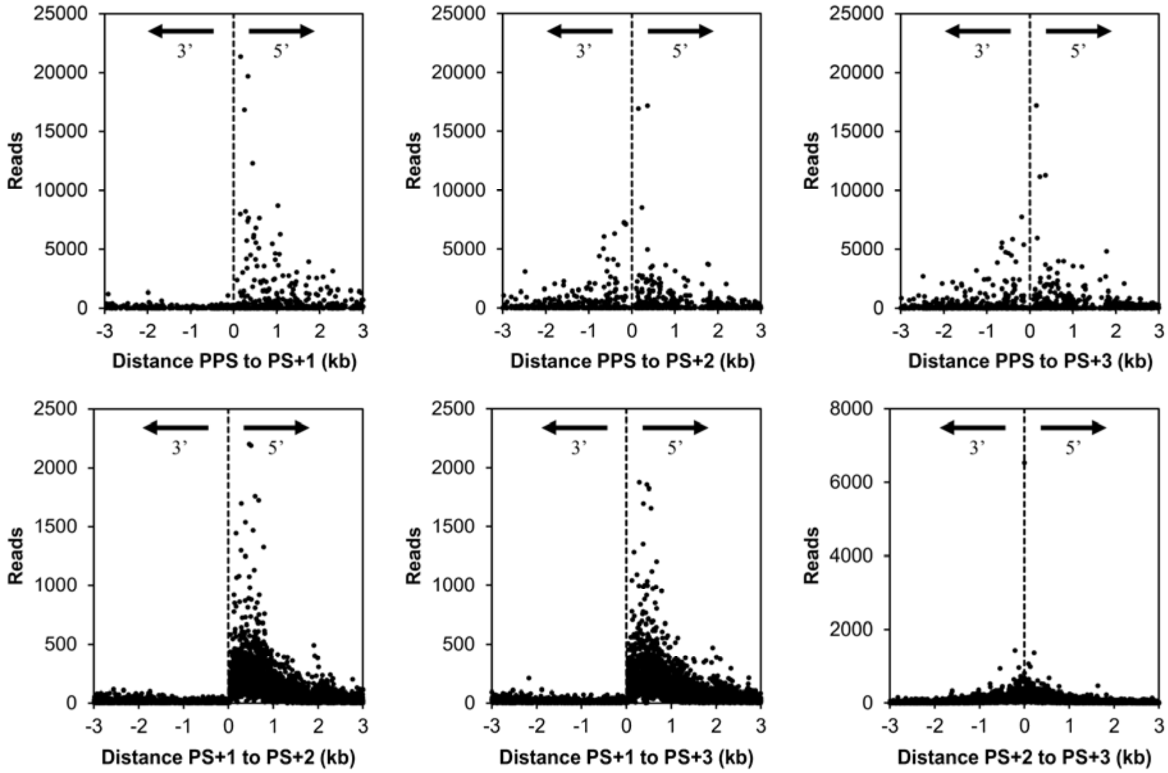

Supplementary Figure 7 | Relationship between the first acquired spacer and subsequent spacer positions during priming. Read-weighted distances between plasmid mapping protospacer locations for the pPriming(-) dataset, created for subsequent protospacers on the non-primed (PS+1), primed (PS+2 or PS+3) strands. Positive distances represent 5’ travel, and - because the plasmid is circular - the shortest travel between two locations was used.

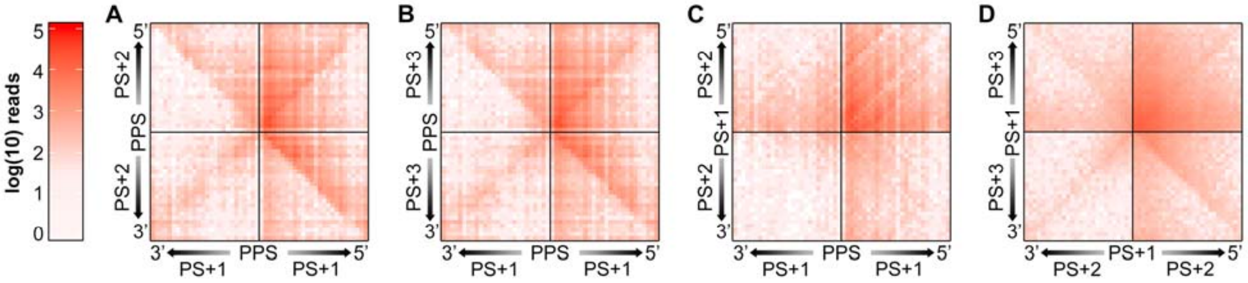

Supplementary Figure 8 | Directionality bias of sequentially acquired spacers (irrespective of which strand was targeted). Heatmaps (similar to Fig. 6) created for the ‘travel distances’ in the pPriming(-) dataset, irrespective of which strands the subsequent protospacers (PS+1, PS+2 and PS+3) were located on. **(a)** PPS to PS+1 and PPS to PS+2, **(b)** PPS to PS+1 and PPS to PS+3, **(c)** PPS to PS+1 and PS+1 to PS+2, and **(d)** PS+1 to PS+2 and PS+1 to PS+3. These plots demonstrate the strength of the influence that the first spacer acquired has over subsequent spacer acquisitions. At first sight it might appear surprising that PS+1 exerts such a strong influence over all subsequent acquisitions, but that PS+2 and beyond do not. However, this finding fits with the biochemical mechanisms underlying the adaptation process. Before a newly captured (proto)spacer can stimulate acquisition of subsequent spacers it must first be inserted into a CRISPR array, expressed, processed and packaged with the Csy proteins to form an active surveillance complex – meanwhile existing spacers, already represented in the surveillance pool, are free to elicit their effects. Hence, PS+2 is many steps behind PS+1, and we theorise that this temporal gap explains why PS+1 exerts more influence over subsequent interference-driven acquisitions than PS+2 or beyond. It is indeed likely that PS+2 and beyond can also trigger interference-driven acquisition of new spacers. However, the overall contribution of this to our dataset appears low, possibly because the plasmids might already have been degraded by the time these crRNAs are loaded onto Csy complexes.

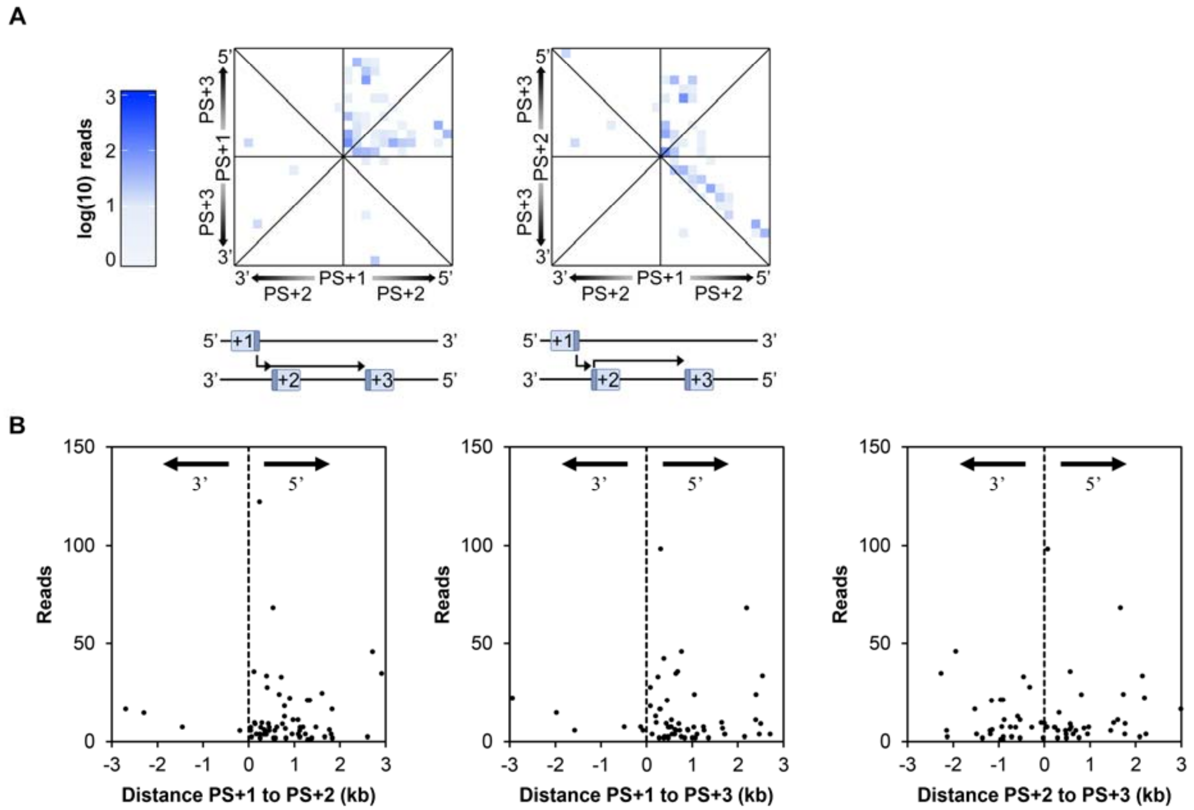

Supplementary Figure 9 | Relationship between the first acquired protospacer and subsequent protospacer positions during naive acquisition. (a) Heatmaps created for the 'travel distances' in the pNaive dataset for the subgroups where PS+2 and PS+3 were acquired from the opposite strand to PS+1 (i.e. plus-minus-minus and minus-plus-plus). **(b)** Read-weighted average distances between plasmid mapping protospacer locations for the pNaive subgroup as in (a). Positive distances represent 5' travel, and because the plasmid is circular, the shortest travel between two locations was used.

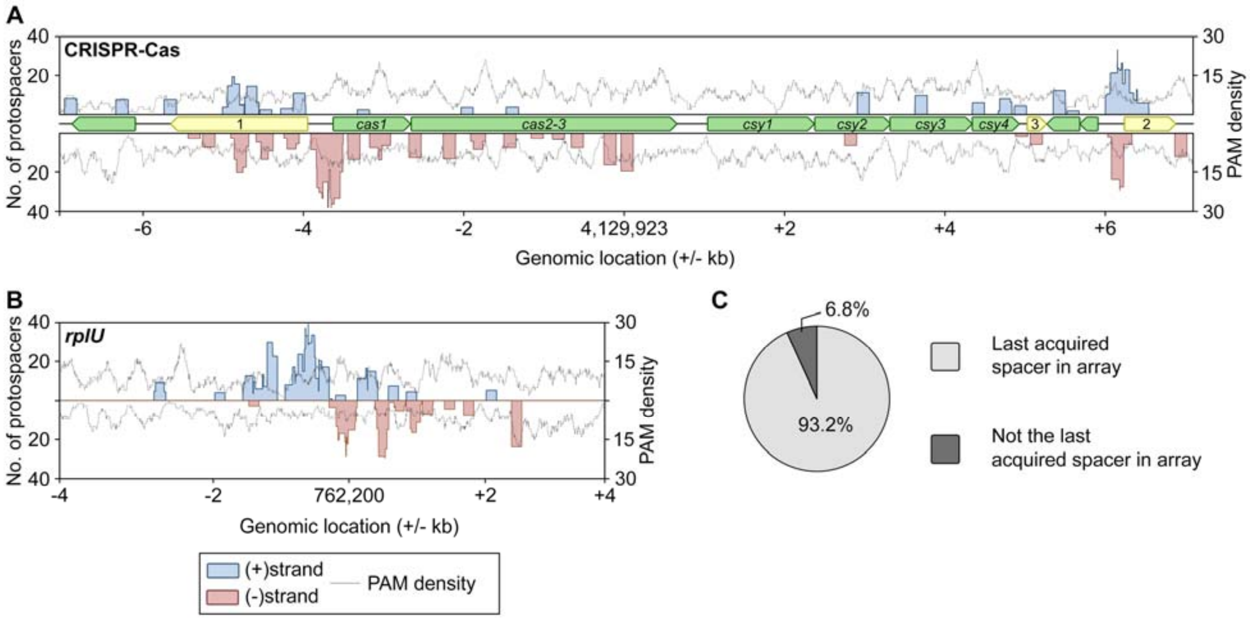

Supplementary Figure 10 | DNA breaks and highly transcribed regions result in chromosomal spacer acquisition. The protospacer locations were mapped on the **(a)** type I-F CRISPR-Cas and **(b)** *rplU* regions using a sliding 150 nt binning window. Protospacers on the plus and minus strand are indicated in blue and red, respectively. The dotted gray graph in the background depicts the PAM distribution, which is the density of PAM sequences across the plasmid using a sliding 150 nt binning window. In (a) genes (green) and CRISPR arrays (yellow) on the chromosome are schematically depicted between the plus and minus graphs. **(c)** Proportion of arrays in which any chromosomal-targeting spacer was the last spacer acquired or was elsewhere in the CRISPR array.

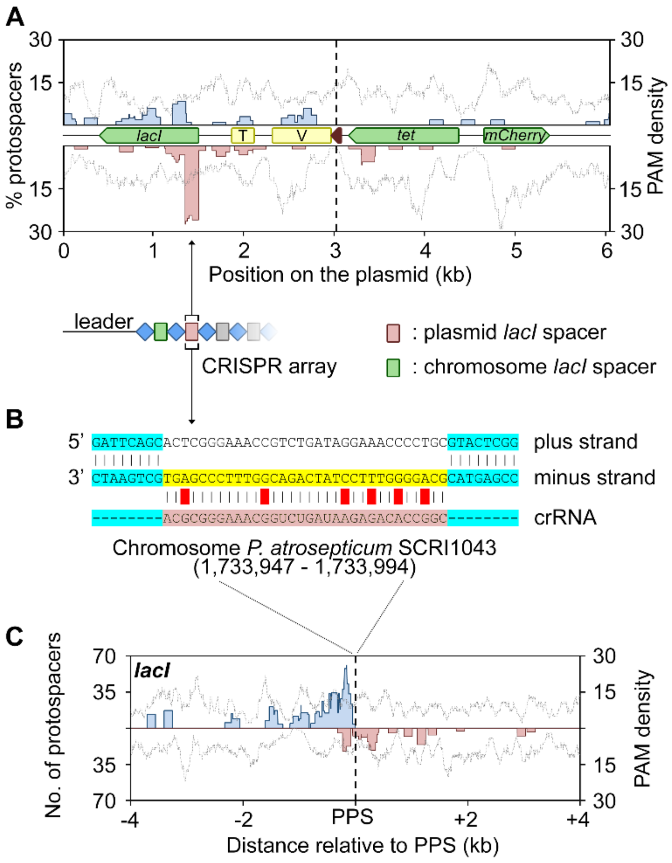

Supplementary Figure 11 | Primed acquisition of *P. atrosepticum lacI*-targeting chromosomal spacers is stimulated by acquisition of a spacer from the plasmid *E. coli lacI* gene. (a) Arrays containing spacers targeting *lacI* on the chromosome were analyzed for the location of preceding protospacers that were derived from the plasmid. Protospacer locations were mapped on pPriming(-) using a sliding 150 nt binning window. **(b)** Approximately 50% of arrays contained a plasmid-derived spacer that mapped to the *P. atrosepticum lacI* gene with 6 mismatches and a non-consensus PAM (CRISPRTarget output). **(c)** The protospacer locations were mapped on the chromosomal *lacI* region using a sliding 150 nt binning window. In (a) and (c) labeling is the same as Fig. 4.

Supplementary Table 1 | Strains and plasmids used in this study.

Strain	Genotype	Reference
Pectobacterium atrosepticum SCRI1043	Wild-type (WT)	3
Escherichia coli	ST18	4

Plasmids	Description	Reference
pNaive	Identical to pPF571: pQE-80LoriT-mCherry-derivative, Tc^R	5
pPriming(-)	Identical to pPF575: pQE-80LoriT-mCherry-derivative, contains a priming protospacer on the minus strand complementary to spacer 1 from CRISPR1, Tc^R	5
pPriming(+)	Identical to pPF574: pQE-80LoriT-mCherry-derivative, contains a priming protospacer on the plus strand complementary to spacer 1 from CRISPR1, Tc^R	5
pTargeted(-)	Similar to pPriming(-), but contains a targeted protospacer (GG PAM) on the minus strand complementary to spacer 1 from CRISPR1, Tc^R	This study
pNaive:ACR	Based on pNaive, with addition of AraC and the *Delftia* sp. 670 AcrF8 anti-CRISPR under control of P_{araBAD}.	This study
pNaive:Control	Based on pNaive, with addition of AraC and P_{araBAD} but no anti-CRISPR ORF.	This study
pTargeted(-):ACR	Based on pTargeted(-), with addition of AraC and the *Delftia* sp. 670 AcrF8 anti-CRISPR under control of P_{araBAD}.	This study
pTargeted(-):Control	Based on pTargeted(-), with addition of AraC and P_{araBAD} but no anti-CRISPR ORF.	This study
pPriming(-):ACR	Based on pPriming(-), with addition of AraC and the *Delftia* sp. 670 AcrF8 anti-CRISPR under control of P_{araBAD}.	This study
pPriming(-):Control	Based on pPriming(-), with addition of AraC and P_{araBAD} but no anti-CRISPR ORF.	This study

Supplementary Table 2 | Primers used in this study.

**Primer name**	**Sequence** (5’-3’)*	**Description**
PF174	CGTTAGAGTGATCGGGCTAC	F CRISPR1 leader
PF175	CAATGGCTCAGGGGATTC	R CRISPR1 spacer2
PF1461	acaaccCGTTAGAGTGATCGGGCTAC	F CRISPR1 leader pNaive #1
PF1462	ttaaccCGTTAGAGTGATCGGGCTAC	F CRISPR1 leader pNaive #2
PF1463	cgcgtcCGTTAGAGTGATCGGGCTAC	F CRISPR1 leader pNaive #3
PF1464	caagagAACTACCGTAAAATAGGAACGG	F CRISPR2 leader pNaive #1
PF1465	tctaatAACTACCGTAAAATAGGAACGG	F CRISPR2 leader pNaive #2
PF1466	atagcaAACTACCGTAAAATAGGAACGG	F CRISPR2 leader pNaive #3
PF1467	tcgacgTGTTAGTGTGAAAATTTAACAGTTC	F CRISPR3 leader pNaive #1
PF1468	atgattTGTTAGTGTGAAAATTTAACAGTTC	F CRISPR3 leader pNaive #2
PF1469	taagaaTGTTAGTGTGAAAATTTAACAGTTC	F CRISPR3 leader pNaive #3
PF1470	ctcaggGGGATTCTACAACCCTAATTTC	R CRISPR1 spacer2 pNaive #1
PF1471	caacacGGGATTCTACAACCCTAATTTC	R CRISPR1 spacer2 pNaive #2
PF1472	agaactGGGATTCTACAACCCTAATTTC	R CRISPR1 spacer2 pNaive #3
PF1473	tggtcgCTTTAAGCGCATGTCGGTC	R CRISPR2 spacer2 pNaive #1
PF1474	acgtaaCTTTAAGCGCATGTCGGTC	R CRISPR2 spacer2 pNaive #2
PF1475	tccggtCTTTAAGCGCATGTCGGTC	R CRISPR2 spacer2 pNaive #3
PF1476	acgcatCAGAAAGCCGACTTCAATC	R CRISPR3 spacer2 pNaive #1
PF1477	cattacCAGAAAGCCGACTTCAATC	R CRISPR3 spacer2 pNaive #2
PF1478	cgtgagCAGAAAGCCGACTTCAATC	R CRISPR3 spacer2 pNaive #3
PF1479	tggcatCGTTAGAGTGATCGGGCTAC	F CRISPR1 leader pPriming(-) #1
PF1480	taggatCGTTAGAGTGATCGGGCTAC	F CRISPR1 leader pPriming(-) #2
PF1481	accataCGTTAGAGTGATCGGGCTAC	F CRISPR1 leader pPriming(-) #3
PF1482	attatcAACTACCGTAAAATAGGAACGG	F CRISPR2 leader pPriming(-) #1
PF1483	ccacaaAACTACCGTAAAATAGGAACGG	F CRISPR2 leader pPriming(-) #2
PF1484	ttaattAACTACCGTAAAATAGGAACGG	F CRISPR2 leader pPriming(-) #3
PF1485	tcctcaTGTTAGTGTGAAAATTTAACAGTTC	F CRISPR3 leader pPriming(-) #1
PF1486	tagaagTGTTAGTGTGAAAATTTAACAGTTC	F CRISPR3 leader pPriming(-) #2
PF1487	ctgacgTGTTAGTGTGAAAATTTAACAGTTC	F CRISPR3 leader pPriming(-) #3
PF1488	aggagcGGGATTCTACAACCCTAATTTC	R CRISPR1 spacer2 pPriming(-) #1
PF1489	cacacgGGGATTCTACAACCCTAATTTC	R CRISPR1 spacer2 pPriming(-) #2
PF1490	ccgacaGGGATTCTACAACCCTAATTTC	R CRISPR1 spacer2 pPriming(-) #3
PF1491	ttacgaCTTTAAGCGCATGTCGGTC	R CRISPR2 spacer2 pPriming(-) #1
PF1492	agattaCTTTAAGCGCATGTCGGTC	R CRISPR2 spacer2 pPriming(-) #2
PF1493	ctccgtCTTTAAGCGCATGTCGGTC	R CRISPR2 spacer2 pPriming(-) #3
PF1494	atgcaaCAGAAAGCCGACTTCAATC	R CRISPR3 spacer2 pPriming(-) #1
PF1495	aaggccCAGAAAGCCGACTTCAATC	R CRISPR3 spacer2 pPriming(-) #2
PF1496	acgagaCAGAAAGCCGACTTCAATC	R CRISPR3 spacer2 pPriming(-) #3
PF1497	ccgtgtCGTTAGAGTGATCGGGCTAC	F CRISPR1 leader pPriming(+) #1
PF1498	cacaacCGTTAGAGTGATCGGGCTAC	F CRISPR1 leader pPriming(+) #2
PF1499	tcatatCGTTAGAGTGATCGGGCTAC	F CRISPR1 leader pPriming(+) #3
PF1500	acctgtAACTACCGTAAAATAGGAACGG	F CRISPR2 leader pPriming(+) #1
PF1501	cggaagAACTACCGTAAAATAGGAACGG	F CRISPR2 leader pPriming(+) #2
PF1502	ttcggcAACTACCGTAAAATAGGAACGG	F CRISPR2 leader pPriming(+) #3
PF1503	aaggttTGTTAGTGTGAAAATTTAACAGTTC	F CRISPR3 leader pPriming(+) #1
PF1504	ttctccTGTTAGTGTGAAAATTTAACAGTTC	F CRISPR3 leader pPriming(+) #2
PF1505	ccaataTGTTAGTGTGAAAATTTAACAGTTC	F CRISPR3 leader pPriming(+) #3
PF1506	aagtatGGGATTCTACAACCCTAATTTC	R CRISPR1 spacer2 pPriming(+) #1
PF1507	cggagtGGGATTCTACAACCCTAATTTC	R CRISPR1 spacer2 pPriming(+) #2

PF1508	tatcggGGGATTCTACAACCCTAATTTC	R CRISPR1 spacer2 pPriming(+) #3
PF1509	tgccgcCTTTAAGCGCATGTCGGTC	R CRISPR2 spacer2 pPriming(+) #1
PF1510	tccattCTTTAAGCGCATGTCGGTC	R CRISPR2 spacer2 pPriming(+) #2
PF1511	aagtcgCTTTAAGCGCATGTCGGTC	R CRISPR2 spacer2 pPriming(+) #3
PF1512	ccacgcCAGAAAGCCGACTTCAATC	R CRISPR3 spacer2 pPriming(+) #1
PF1513	tcctggCAGAAAGCCGACTTCAATC	R CRISPR3 spacer2 pPriming(+) #2
PF1514	tgtctaCAGAAAGCCGACTTCAATC	R CRISPR3 spacer2 pPriming(+) #3
PF1763	AACGACTAGTGGTAATGACTCTCTAGCTTGAGGC	Plasmid forward with SpeI site
PF1764	ACCACTAGTCGTTCTGATTTAATCTGTATCAGG	pBAD30 reverse with SpeI site
PF1765	CATGCGGCCGCGATCTGCTCATGTTTGACAGC	pBAD30 forward with NotI site
PF1766	ATCGCGGCCGCATGATGAACCTGAATCGCCAG	Plasmid reverse with NotI site

*For primers PF1461 to PF1514, the 6 nucleotide barcodes are indicated with lower case letters.

Supplementary References

1. Biswas, A., Staals, R. H., Morales, S. E., Fineran, P. C. & Brown, C. M. CRISPRDetect: A flexible algorithm to define CRISPR arrays. *BMC Genomics* **17**, 356, doi:10.1186/s12864-016-2627-0 (2016).
2. Biswas, A., Gagnon, J. N., Brouns, S. J., Fineran, P. C. & Brown, C. M. CRISPRTarget: bioinformatic prediction and analysis of crRNA targets. *RNA biology* **10**, 817-827, doi:10.4161/rna.24046 (2013).
3. Bell, K. S. *et al.* Genome sequence of the enterobacterial phytopathogen Erwinia carotovora subsp. atroseptica and characterization of virulence factors. *Proceedings of the National Academy of Sciences of the United States of America* **101**, 11105-11110, doi:10.1073/pnas.0402424101 (2004).
4. An improved *Escherichia coli* donor strain for diparental mating. *FEMS Microbiol Lett.* **294**, 127-132, doi: 10.1111/j.1574-6968.2009.01556.x (2009).
5. Richter, C. *et al.* Priming in the Type I-F CRISPR-Cas system triggers strand-independent spacer acquisition, bi-directionally from the primed protospacer. *Nucleic Acids Res* **42**, 8516-8526, doi:10.1093/nar/gku527 (2014).
